# VolGAN: A Generative Model for Arbitrage-Free Implied Volatility Surfaces

**DOI:** 10.1080/1350486X.2025.2471317

**Published:** 2025-03-06

**Authors:** Milena Vuletić, Rama Cont

**Affiliations:** Mathematical Institute, University of Oxford, Oxford, UK

**Keywords:** Generative models, GenAI, scenario simulation, volatility surface, options, implied volatility

## Abstract

We introduce VolGAN, a generative model for arbitrage-free implied volatility surfaces. The model is trained on time series of implied volatility surfaces and underlying prices and is capable of generating realistic scenarios for joint dynamics of the implied volatility surface and the underlying asset. We illustrate the performance of the model by training it on SPX implied volatility time series and show that it is able to learn the covariance structure of the co-movements in implied volatilities and generate realistic dynamics for the (VIX) volatility index. In particular, the generative model is capable of simulating scenarios with non-Gaussian distributions of increments for state variables as well as time-varying correlations. Finally, we illustrate the use of VolGAN to construct data-driven hedging strategies for option portfolios, and show that these strategies can outperform Black–Scholes delta and delta-vega hedging.

## 1. Introduction

Option prices are quoted in terms of their *implied volatilities*, which are obtained by inverting the Black–Scholes formula given the market prices of options. The implied volatility surface, which summarizes the cross-section of option prices across strikes and maturities, gives a snapshot of the state of the options market. The dependence of implied volatility on moneyness and time-to-maturity, which is referred to as the *smile*, *skew* and *term structure* have inspired the development of alternative option pricing models (Cont and Tankov 2004; Gatheral 2011; Heston 1993). Any such option pricing model implies a model for the cross-sectional dependence of implied volatilities on strike and maturity, as well as their dynamics across time. However, this dynamics is typically intractable and there has been an interest from practitioners in directly modelling the dynamics of implied volatility as a state variable (Avellaneda et al. 2020; Babbar 2001; Cont and da Fonseca 2002; Cont, Fonseca, and Durrleman 2002; Cont and Vuletic 2023; Durrleman 2010; Schönbucher 1999). Such 'market models' of implied volatility should appropriately capture the co-movements of implied volatilities across moneyness and time-to-maturity, reproduce the empirically observed dynamics of implied volatilities (Cont and da Fonseca 2002), be able to capture the smile, skew, and term structure, and satisfy arbitrage constraints (Davis and Hobson 2007; Gerhold and Gülüm 2020).

Given the high dimensionality of the volatility surface and the complexity of its dynamics, it is challenging to capture all these properties in a parametric model. It is therefore of interest to examine whether a data-driven approach can be used to overcome these modelling challenges.

### 1.1. Contribution

In the present work we introduce VolGAN, a *fully data-driven* generative model for the dynamic simulation of arbitrage-free implied volatility surfaces. Our model is trained on a time series of market-quoted implied volatilities and is capable of generating realistic dynamic scenarios for implied volatility surfaces. We illustrate the performance of the model by training it on SPX implied volatility time series and show that it is able to learn the covariance structure of co-movements in implied volatilities and generate realistic dynamics for the (VIX) volatility index (CBOE 2022). In particular, the generative model is capable of simulating scenarios with non-Gaussian distributions of increments for state variables as well as time-varying correlations.

Last but not least, we show that VolGAN may be used to compute data-driven hedging strategies for option porfolios. Using examples of SPX option portfolios, we show that VolGAN can produce hedge ratios with better performance than Black–Scholes delta hedging and delta-vega hedging, with automatic selection of the hedging instruments. In contrast with model-based approaches such as Deep hedging (Buehler et al. 2019), our approach is completely *data-driven* and model-free, in the spirit of the pioneering work of Hutchinson, Lo, and Poggio (1994).

Our model builds on previous work on generative adversarial networks (GANs) for scenario simulation in finance, starting with Takahashi, Chen, and Tanaka-Ishii (2019) and Wiese et al. (2020) for price dynamics. More recently, GAN methods have been deployed for scenario simulation in options markets. Wiese et al. (2019) uses a classical GAN approach. Cuchiero, Khosrawi, and Teichmann (2020) and Cohen, Reisinger, and Wang (2022) use a ‘neural SDE’ to parameterize volatility surface dynamics. Cao, Chen, and Hull (2020) use a supervised learning approach to extract information from historical implied volatility dynamics, while Ning et al. (2023) combines SDEs with Variational Autoencoders (Kingma and Welling 2019).

In contrast with the aforementioned approaches which deploy the classical GAN methodology of Goodfellow et al. (2014) using binary cross-entropy (BCE) as a training objective, we propose a bespoke training criterion adapted to the financial application at hand, as advocated in Cont et al. (2022) and Vuletić, Prenzel, and Cucuringu (2024), combined with a scenario weighting approach based on Cont and Vuletic (2023) to take care of arbitrage constraints.

### 1.2. Outline

Section 2 summarizes properties of implied volatility surfaces and outlines some desirable requirements for a dynamic model of implied volatility. Section 3 describes VolGAN, our proposed generative model for implied volatility surfaces. Section 4 presents the results obtained by training VolGAN on SPX implied volatility data and discusses the model's ability to produce realistic scenarios for implied volatility co-movements and the VIX index. Section 5 demonstrates applications of VolGAN for hedging and shows that hedging strategies computed using VolGAN can outperform commonly used delta hedging and delta-vega hedging strategies.

## 2. Implied Volatility Surfaces: Shape Constraints and Dynamics

Denoting the price of the underlying asset by $S_{t}$, the implied volatility may be parameterized in terms of moneyness $m=K/S_{t}$ and time to maturity τ=T−t of the option. The implied volatility associated with a call option with moneyness *m* and time-to-maturity *τ* on a non-dividend paying asset *S* is the unique value $\sigma_{t}(m,\tau )$ such that the Black–Scholes price $C_{\mathrm{BS}}(S_{t},K,\tau ,\sigma_{t}(m,\tau ))$ matches the market price $C_{t}(m,\tau )$ of the call:

$$\begin{array}{rl} C_{t}(m,\tau ) & =C_{\mathrm{BS}}(S_{t},K,\tau ,\sigma_{t}(m,\tau ))=S_{t}N(d_{1})-Ke^{-\mathrm{r\tau}}N(d_{2})\\ d_{1} & =\frac{-\ln m+\tau (r+\frac{\sigma^{2}}{2})}{\sigma \sqrt{\tau }}\;d_{2}=\frac{-\ln m+\tau (r-\frac{\sigma^{2}}{2})}{\sigma \sqrt{\tau }}, \end{array}$$

where *N* is the c.d.f of a standard Gaussian N(0,1) variable. The implied volatility surface $\sigma_{t}(m,\tau )$ at date *t* provides a snapshot of options prices in the market (Gatheral 2011): specifying the implied volatility surface is equivalent to specifying the prices of all European calls and puts available in the market, given the current term structure of interest rates and dividends.

### 2.1. Static Arbitrage and Shape Constraints

It has been empirically observed that implied volatilities of call and put options in listed options markets exhibit a dependence on exercise price *K* and maturity date *T* (Cont and da Fonseca 2002; Dumas, Fleming, and Whaley 1998; Dupire 1994; Gatheral 2011) (or, alternatively, on the moneyness $m=K/S_{t}$ and time-to-maturity τ=T−t). However not every cross-sectional profile for the function $\left(m,\tau )\mapsto \sigma_{t}(m,\tau \right)$ is admissible, as the resulting call/put option prices should satisfy certain *static arbitrage constraints* (Davis and Hobson 2007; Gerhold and Gülüm 2020). In particular call option prices should be:

- increasing in time to maturity: $\partial_{\tau }C_{\mathrm{BS}}(S_{t},K,\tau ,\sigma_{t}(m,\tau ))\geq 0$,
- decreasing in moneyness: $\partial_{m}C_{\mathrm{BS}}(S_{t},K,\tau ,\sigma_{t}(m,\tau ))\leq 0,$
- convex in moneyness: $\partial_{m}^{2}C_{\mathrm{BS}}(S_{t},K,\tau ,\sigma_{t}(m,\tau ))\geq 0$.

These constraints translate to nonlinear inequalities involving $\sigma_{t}$, $\partial_{m}\sigma_{t}$, $\partial_{m}^{2}\sigma_{t}$, $\partial_{\tau }\sigma_{t}$ (Cont, Fonseca, and Durrleman 2002), which in turn impose constraints on the possible shapes of the implied volatility surface $\sigma_{t}(m,\tau )$.

Given a fixed grid in moneyness and time to maturity

$$(m,\tau )=(m_{i},\tau_{j}{)}_{i=1,\ldots ,N_{m};j=1,\ldots N_{\tau }},$$

with $m_{i}<m_{i+1}$ and $\tau_{j}<\tau_{j+1}$, we define the relative call prices

(1) $$c(m,\tau ):=\frac{1}{S}C_{\mathrm{BS}}(S,K,\tau ,\sigma )=N(d_{1})-me^{-\mathrm{r\tau}}N(d_{2}).$$

Following Cont and Vuletic (2023), we define the *arbitrage penalty* associated with the (discretely sampled) volatility surface σ(m,τ) as:

(2) $$\Phi \left(\sigma (m,\tau )\right)=p_{1}(\sigma (m,\tau ))+p_{2}(\sigma (m,\tau ))+p_{3}(\sigma (m,\tau )).$$

where the functions $p_{1},p_{2},p_{3}$ measure violations of calendar, call and butterfly arbitrage constraints, respectively:

(3) $$\begin{array}{rl} p_{1}(\sigma (m,\tau )) & =\sum_{i=1}^{N_{m}}\sum_{j=1}^{N_{\tau }}{\left(\tau_{j}\frac{c(m_{i},\tau_{j})-c(m_{i},\tau_{j+1})}{\tau_{j+1}-\tau_{j}}\right)}^{+}, \end{array}$$

(4) $$\begin{array}{rl} p_{2}(\sigma (m,\tau )) & =\sum_{i=1}^{N_{m}}\sum_{j=1}^{N_{\tau }}{\left(\frac{c(m_{i+1},\tau_{j})-c(m_{i},\tau_{j})}{m_{i+1}-m_{i}}\right)}^{+}, \end{array}$$

(5) $$\begin{array}{rl} p_{3}(\sigma (m,\tau )) & =\sum_{i=1}^{N_{m}}\sum_{j=1}^{N_{\tau }}{\left(\frac{c(m_{i},\tau_{j})-c(m_{i-1},\tau_{j})}{m_{i}-m_{i-1}}-\frac{c(m_{i+1},\tau_{j})-c(m_{i},\tau_{j})}{m_{i+1}-m_{i}}\right)}^{+}. \end{array}$$

Static arbitrage constraints (Davis and Hobson 2007) are then equivalent to

Φ(σ(m,τ))=0

and the magnitude of Φ(σ(m,τ)) can be considered as a ‘distance’ from the set of arbitrage-free implied volatility surfaces.

### 2.2. Dynamics of Implied Volatility Co-movements

Static arbitrage constraints on the shape of the implied volatility surface are a necessary but not sufficient requirement for a good model of implied volatility dynamics: one also needs the model to capture the statistical properties of implied volatility co-movements, a crucial point for any hedging and risk management task. Here we summarize some of the empirically observed statistical properties of implied volatilities on various exchange-traded indices (Avellaneda et al. 2020; Cont and da Fonseca 2002; Cont and Vuletic 2023):

- The implied volatility has a non-flat cross-section, with dependence in strike and maturity.
- Implied volatilities display high positive autocorrelation and mean-reverting behaviour.
- Daily variations in the implied volatilities can be satisfactorily explained with a small number of principal components.
- The first principal component corresponds to a *level*, whereas the second principal component corresponds to a *skew* factor.
- The returns of the underlying are negatively correlated with the projections of log-increments of implied volatility on the *level* and *skew* principal components, which is a more precise formulation of the so-called 'leverage effect'.

We now describe a data-driven approach for the simulation of implied volatility dynamics designed to account for the above properties.

## 3. A Generative Model for Implied Volatility Surfaces

VolGAN is a customized conditional generative adversarial network with a smoothness penalty incorporated into the generator's loss function, combined with scenario re-weighting applied to the output scenarios (Cont and Vuletic 2023).

VolGAN receives as input

- the implied volatility surface at the previous date,
- the two previous underlying returns,
- the realized volatility from the previous period,

and outputs (joint) scenarios for

- the return of the underlying asset and
- the implied volatility surface

for the next period, along with a set of weights (probabilities) associated with these scenarios. We now discuss the methodology in more detail.

### 3.1. Architecture

We design a Conditional GAN (Mirza and Osindero 2014), composed of two neural networks, a *generator* and a *discriminator*. Suppose we have observations at times t∈T, in increments of Δt=1/252 (1 day), with $S_{t}$ the price of the underlying, and $\sigma_{t}(m,\tau )$ the implied volatility surface on the grid (m,τ) at time *t*. Denote by $g_{t}(m,\tau )$ the log-implied volatility surface at time *t*:

(6) $$g_{t}(m,\tau )=\log\sigma_{t}(m,\tau ),\;\Delta g_{t}(m,\tau )=g_{t+\mathrm{\Delta t}}(m,\tau )-g_{t}(m,\tau ).$$

Let $R_{t}$ be the log-return of the underlying:

(7) $$R_{t}=\log\left(\frac{S_{t+\mathrm{\Delta t}}}{S_{t}}\right),$$

and denote by $\gamma_{t}$ the one-month realized volatility:

(8) $$\gamma_{t}=\sqrt{\frac{252}{21}\sum_{i=0}^{20}R_{t-i\mathrm{\Delta t}}^{2}}.$$

We aggregate $R_{t-\mathrm{\Delta t}},R_{t-2\mathrm{\Delta t}},\gamma_{t-\mathrm{\Delta t}},g_{t}(m,\tau )$ into a *condition*/*input* vector $a_{t}$:

(9) $$a_{t}=(R_{t-\mathrm{\Delta t}},R_{t-2\mathrm{\Delta t}},\gamma_{t-\mathrm{\Delta t}},g_{t}(m,\tau )).$$

The generator G takes as input this condition $a_{t}$ and i.i.d. noise $z_{t}∼N(0,I_{d})$ and outputs simulated values $\hat{R_{t}}(z),\Delta \hat{g_{t}}(m,\tau )$ for the return and implied volatility (log-)increments:

(10) $$G(a_{t},z_{t})=(\hat{R_{t}}(z_{t}),\Delta \hat{g_{t}}(m,\tau )(z_{t})).$$

We denote by $G(a_{t},z){|}_{2:}=\Delta \hat{g_{t}}(m,\tau )(z)$ the second component of the generator's output which corresponds to the simulated log implied volatility increment.

The discriminator is a classifier, taking as input a value (r,Δg) representing either the output of the generator or the corresponding data realization, together with a condition vector $a_{t}$ as in (9). It outputs a value $D(a_{t},(R,\mathrm{\Delta g}))$ between 0 and 1, interpreted as the probability that the input is drawn from the conditional distribution of $\left(R_{t},\Delta g_{t}\right)$ given $a_{t}$.

**Figure 1.**
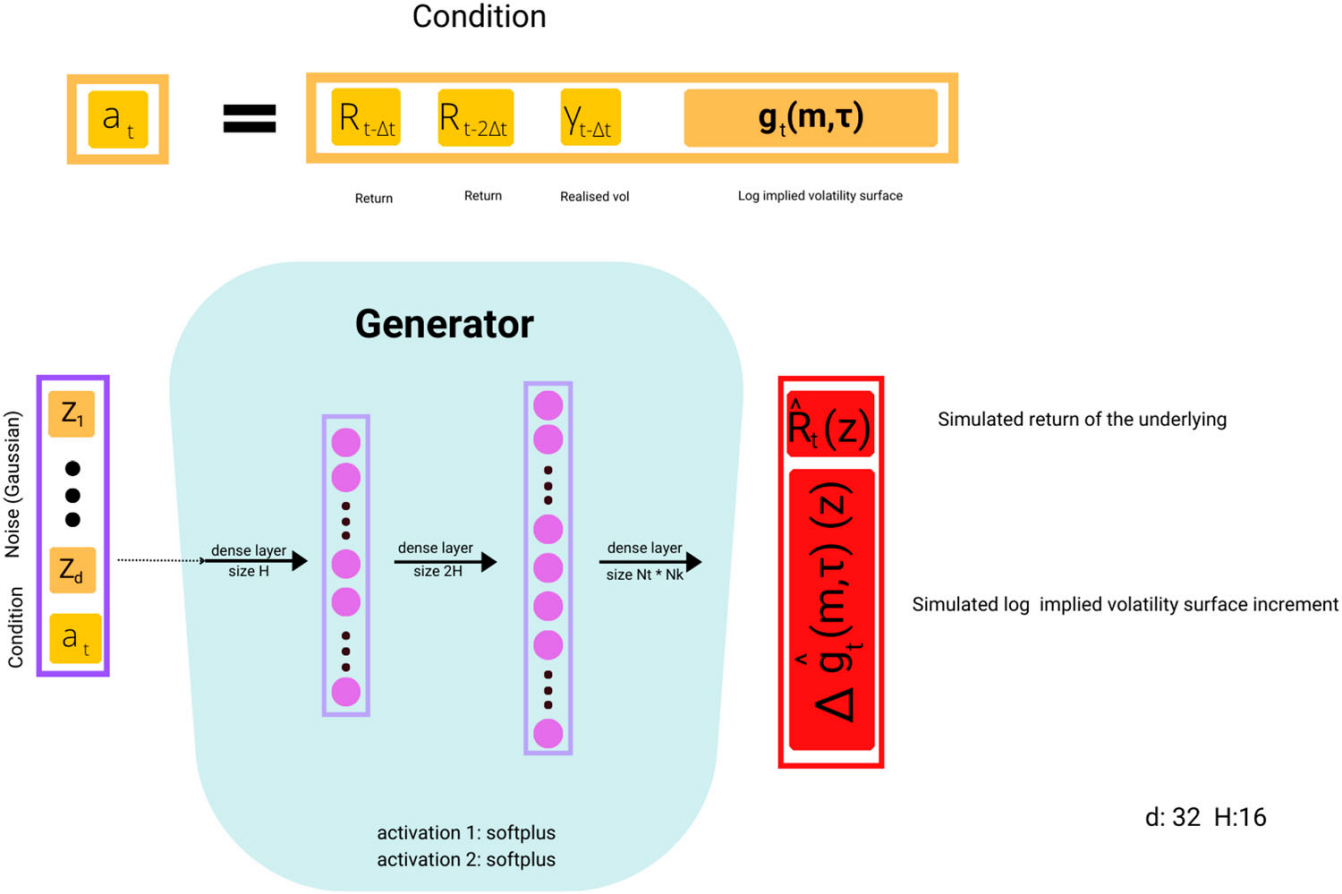
VolGAN generator architecture.

**Figure 2.**
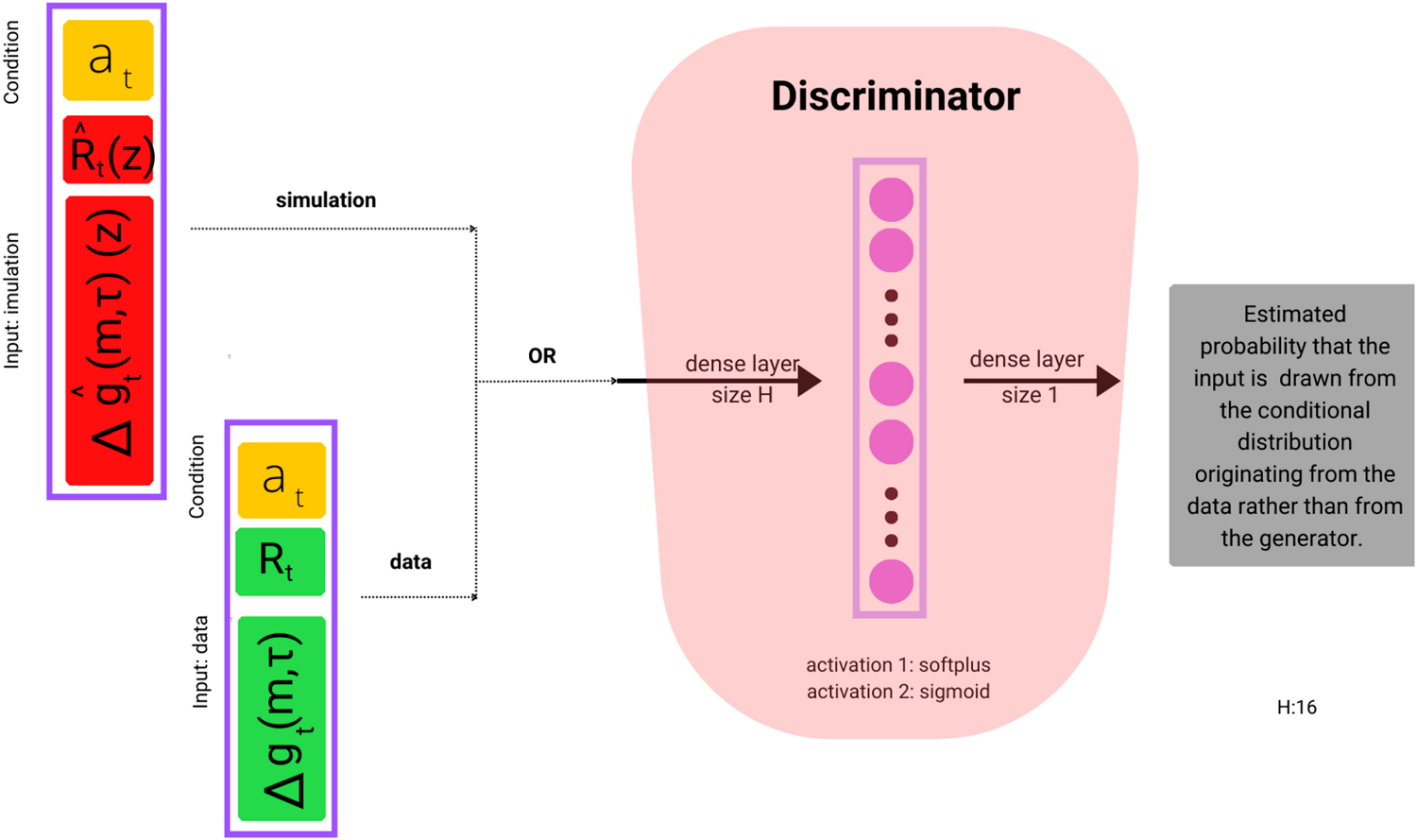
VolGAN discriminator architecture.

The generator *G* and the discriminator *D* are feed-forward neural networks, whose respective parameters (weights) we denote by $\theta_{g}$ and $\theta_{d}$. The architecture of the generator is displayed in Figure 1, and the architecture of the discriminator is shown in Figure 2.

### 3.2. Training Objective

The core component of VolGAN is a customized loss function catering to the desired properties of the output volatility surface, as advocated in Cont et al. (2022). A classical GAN trained using binary cross-entropy (BCE) loss (Goodfellow et al. 2014) would result in irregular surfaces. In order to generate smooth surfaces, we use a smoothness penalty (Jackson, Suli, and Howison 1999; Sana and Cont 2005) defined as a discrete Sobolev semi-norm in *m* and *τ* on the grid (m,τ):

(11) $$\begin{array}{rl} L_{m}(g) & =\sum_{i,j}\frac{{\left(g(m_{i+1},\tau_{j})-g(m_{i},\tau_{j})\right)}^{2}}{{\left|m_{i+1}-m_{i}\right|}^{2}}≃\|\partial_{m}g{\|}_{L^{2}}^{2}, \end{array}$$

(12) $$\begin{array}{rl} L_{\tau }(g) & =\sum_{i,j}\frac{{\left(g(m_{i},\tau_{j+1})-g(m_{i},\tau_{j})\right)}^{2}}{{\left|\tau_{j+1}-\tau_{j}\right|}^{2}}≃\|\partial_{\tau }g{\|}_{L^{2}}^{2}. \end{array}$$

These terms are included in the training objective $J^{\left(G\right)}(\theta_{d},\theta_{g})$ for the generator:

(13) $$\begin{array}{rl} J^{\left(G\right)}(\theta_{d},\theta_{g}) & =-\frac{1}{2}E\left[\log\left(D\left(a_{t},G(a_{t},z_{t};\theta_{g});\theta_{d}\right)\right)\right]\\ & \;+\alpha_{m}E\left[L_{m}\left(g_{t}(m,\tau )+G(a_{t},z_{t};\theta_{g}){|}_{2:}\right)\right]\\ & \;+\alpha_{\tau }E\left[L_{\tau }\left(g_{t}(m,\tau )+G(a_{t},z_{t};\theta_{g}){|}_{2:}\right)\right], \end{array}$$

where $a_{t}=(R_{t-\mathrm{\Delta t}},R_{t-2\mathrm{\Delta t}},\gamma_{t-\mathrm{\Delta t}},g_{t}(m,\tau ))$, as defined in (9). The first term is a binary cross-entropy for the output of the discriminator. $\alpha_{m}>0$ and $\alpha_{\tau }>0$ are regularization parameters, $a_{t}$ is the input ‘condition’ (Equation (9)); $\theta_{g}$ and $\theta_{d}$ are respectively the parameters (weights) of the generator and the discriminator networks. The expectation is computed over the law of the i.i.d. (Gaussian) input $z_{t}∼N(0,I_{d})$. The smoothness penalties $L_{m}$ and $L_{\tau }$ are applied to the simulated log-implied volatility surfaces:

$$g_{t}(m,\tau )+G(a_{t},z_{t};\theta_{g}){|}_{2:}=g_{t}(m,\tau )+\Delta \hat{g_{t}}(m,\tau )(z_{t})=\hat{g_{t}}(m,\tau )(z_{t}).$$

The discriminator is trained to minimize the binary cross-entropy loss:

(14) $$\begin{array}{rl} J^{\left(D\right)}(\theta_{d},\theta_{g}) & =-\frac{1}{2}E\left[\log\left(D(a_{t},(R_{t},\Delta g_{t}(m,\tau ));\theta_{d}\right)\right]\\ & \;-\frac{1}{2}E\left[\log\left(1-D(a_{t},G(a_{t},z_{t};\theta_{g});\theta_{d}\right)\right], \end{array}$$

where $a_{t}$ is the input condition (Equation (9)), $R_{t}$ and $\Delta g_{t}(m,\tau )$ are the corresponding data.

We assume the process $(R_{t},g_{t}{)}_{t\geq 0}$ to be ergodic, so given a long enough sample t∈T we can approximate the expected values above by sample averages:

$$E[f(R_{t},g_{t})]≃\frac{1}{|T|}\sum_{t\in T}f(R_{t},g_{t}).$$

It is possible to incorporate the arbitrage penalty (2) into the loss function of the generator (13). However, we have not done so, and our numerical experiments indicate no notable difference when including it, suggesting that the smoothness penalty is enforcing shape constraints indirectly.

### 3.3. Scenario Re-weighting

The outputs of the generator described above are not guaranteed to satisfy the static arbitrage constraints described in Section 2.1. To correct for this, we apply the methodology described in Cont and Vuletic (2023) to re-weight the one-day-ahead scenarios generated by the GAN.

Let $P_{0}$ be the law of the generator's output i.e., the joint dynamics of the underlying return and the implied volatility surface $\left(R_{t},\sigma_{t}(m,\tau );t\in T\right)$. To adjust for static arbitrage, Cont and Vuletic (2023) apply the change of measure:

(15) $$\frac{dP_{\text{β}}}{dP_{0}}(\omega )=\frac{\exp\left(-\text{β}\Phi (\sigma (m,\tau ;\omega ))\right)}{Z(\text{β})}$$

where Z(β) is a normalization factor:

(16) $$Z(\text{β})=E^{P_{0}}\left[\exp\left(-\text{β}\Phi \left(\sigma (m,\tau ;\omega )\right)\right)\right].$$

VolGAN samples from this target distribution (15) using a Weighted Monte Carlo approach. Given *N* samples from the generator $\left({\hat{R}}^{i},{\hat{\sigma }}^{i}\right)$, i=1,…,N, we compute the arbitrage penalty $\Phi ({\hat{\sigma }}^{i})$ corresponding to each output scenario $\left({\hat{R}}^{i},{\hat{\sigma }}^{i}\right)$ using (2) and sample the scenario $\left({\hat{R}}^{i},{\hat{\sigma }}^{i}\right)$ with probability

(17) $$w^{i}=\frac{\exp(-\text{β}\Phi ({\hat{\sigma }}^{i}))}{\sum_{j=1}^{N}\exp(-\text{β}\Phi ({\hat{\sigma }}^{j}))}.$$

These weighted scenarios may then be used to compute expectations and quantiles of various quantities of interest under $P_{\text{β}}$. Let *X* be a function of the state variables, and let $x_{i}$ be its value in scenario *i*. Denote by $F_{X,\text{β}}$ the law of *X* under $P_{\text{β}}$ and by $E_{\text{β}}[X]$ its expectation. We can estimate $E_{\text{β}}[X]$ by

(18) $$\hat{E_{\text{β}}\left[X\right]}=\sum_{i=1}^{N}w_{i}x_{i},$$

while the quantiles of *X* are estimated as

(19) $$\hat{F_{X,\text{β}}^{-1}(q)}=x_{\left(k\right)},\;\mathrm{where}\;k=\min\left\{j\in \{1,\ldots ,N\}:\sum_{i=1}^{j}w_{\left(i\right)}\geq q\right\},$$

where $x_{\left(1\right)}\leq x_{\left(2\right)}\leq \cdots \leq x_{\left(N\right)}$ are the order statistics of $x_{1},\ldots ,x_{N}$.

### 3.4. Numerical Implementation

The generator *G* is a three-layer feedforward dense neural network, with the first two activations softplus, and the final layer an affine layer. The random input is (standard) i.i.d Gaussian noise with dimension *d* = 32. The first layer consists of *H* = 16 neurons, whereas the second layer contains 2*H* = 32 neurons. Similarly, the discriminator *D* is a two-layer feedforward neural network, with softplus and sigmoidal activation functions and layer sizes of *H* = 16 and 1, respectively. The discriminator has a simpler architecture than the generator, as it is of the utmost importance to keep the two neural networks in balance. The architecture of the discriminator is shown in Figure 2, and the architecture of the generator is displayed in Figure 1.

**Figure 3.**
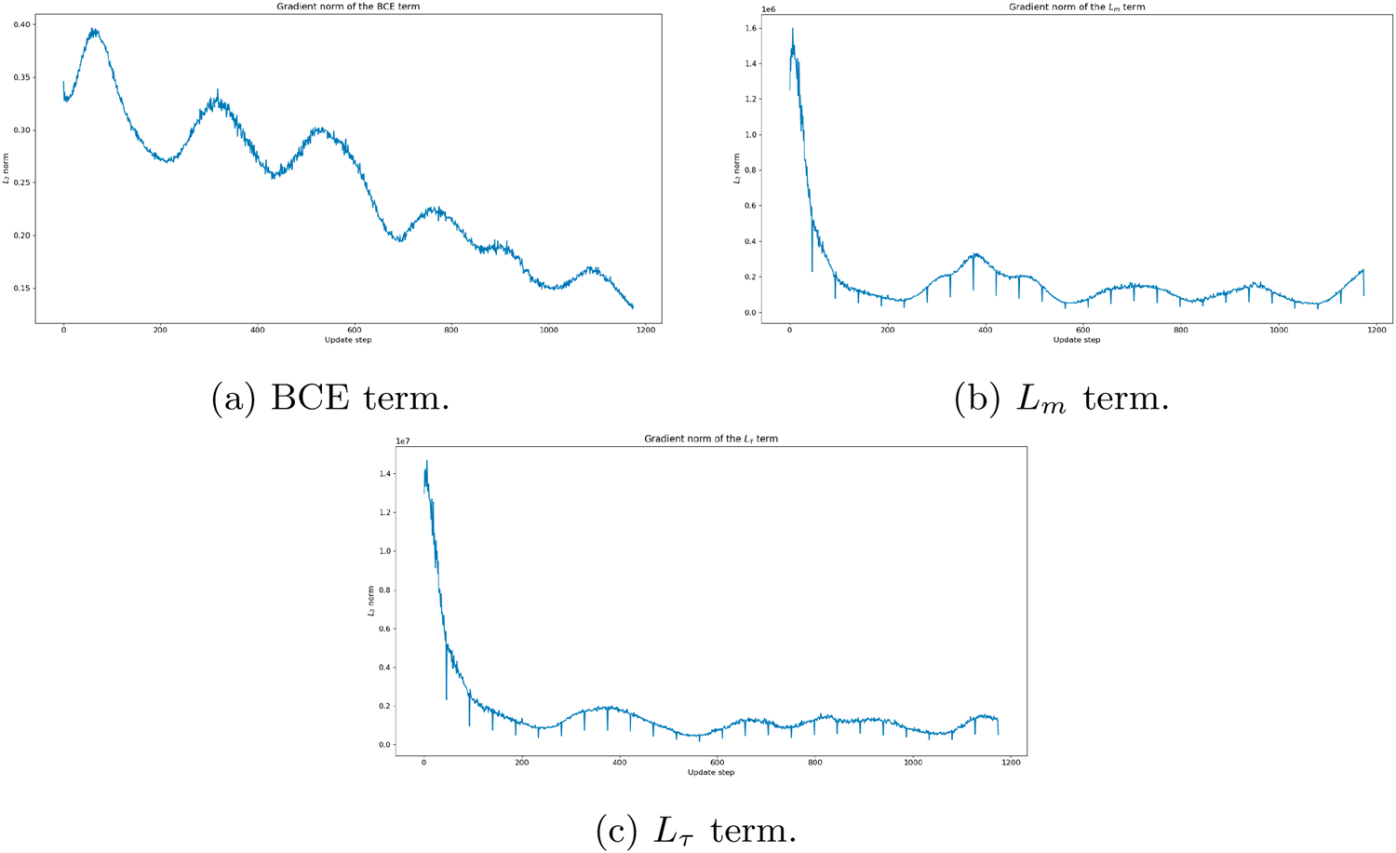
Norm of gradient of the BCE term, $L_{m}$ term, and $L_{\tau }$ term with respect to $\theta_{g}$ during the first stage of VolGAN training: (a) BCE term. (b) $L_{m}$ term and (c) $L_{\tau }$ term.

The hyperparameters $\alpha_{m},\alpha_{\tau }>0$ are chosen by *gradient norm matching*. We first train VolGAN for $n_{\mathrm{grad}}=25$ epochs by performing optimization via the binary cross-entropy loss only (classical GAN setting). At each update, we calculate the gradient norms of each of the three loss function terms in (13): BCE, $L_{m}$, $L_{\tau }$with respect to $\theta_{g}$. We then set $\alpha_{m}$ and $\alpha_{\tau }$, to be the means of observed ratios of the gradient norms of the BCE term to the gradient norms of the $L_{m}$ and $L_{\tau }$, respectively. The gradient norms of the $\mathrm{BCE},L_{m},L_{\tau }$ terms with respect to $\theta_{g}$ during this stage are shown in Figure 3. We note that all three gradients behave similarly, that they stabilize over time, and that there is no gradient explosion or vanishing gradient phenomena.

We then restart training VolGAN (from the same initialization used for the start of the gradient norm matching procedure) with the loss function defined by Equation (13) for $n_{\mathrm{epochs}}=10,000$ epochs, using an alternating direction method i.e., one discriminator update for each generator update. The optimizer used is RMSProp (Hinton, Srivastava, and Swersky 2012), and the learning rates of both networks are set to 0.0001. We take *N* = 10000 raw samples from the generator. The mini-batch size is $n_{\mathrm{batch}}=100$.

#### 3.4.1. Calibration of *β*

The hyperparameter *β* might be chosen by considering the Kullblack–Leibler divergence between the distribution of the weights and the uniform distribution on the scenarios (Cont and Vuletic 2023). Based on the results in Cont and Vuletic (2023), we set

(20) $$\text{β}(t)=\frac{500}{\max\{w_{i}(t)\}},$$

where $w_{i}(t)$ are the weights associated with the generator outputs on day *t*.

## 4. Learning to Simulate SPX Implied Volatility Surfaces

To demonstrate VolGAN's ability to generate realistic scenarios for SPX implied volatility dynamics, we train VolGAN on the daily time series of market data and examine the properties of the generator thus trained. The same approach might be applied to other equity options.

### 4.1. Data

We use the Option Prices file from OptionMetrics. The time period in question is from the 3rd January 2000 to the 28th February 2023, with 3rd Jan 2000-16th Jun 2018 corresponding to the training, and 17th Jun 2019-28th Feb 2023 to the test set. The historical VIX closing prices are available on the CBOE website. The implied risk-free interest rate for each day is calculated as the median rate implied by the put-call parity from the option mid-prices. We construct smooth implied volatility surfaces using the kernel smoothing methodology of Cont and da Fonseca (2002); OptionMetrics (2021). Our grid (m,τ) consists of m∈{0.6,0.7,0.8,0.9,0.95,1,1.05,1.1,1.2,1.3,1.4} and of times to maturity $\tau \in \{\frac{1}{252},\frac{1}{52},\frac{2}{52},\frac{1}{12},\frac{1}{6},\frac{1}{4},\frac{1}{2},\frac{3}{4},1\}$, one day to one year. Suppose that on a fixed day we have available implied volatility data σ(m,τ) for m∈M and τ∈T, with corresponding values of Vega κ(m,τ). We consider a Vega-weighted Nadaraya-Watson kernel smoothing estimator with a 2D Gaussian kernel:

(21) $$\hat{\sigma }(m^{\prime },\tau^{\prime })=\frac{\sum_{m\in M,\tau \in T}\kappa (m,\tau )k(m-m^{\prime },\tau -\tau^{\prime })\sigma (m,\tau )}{\sum_{m\in M,\tau \in T}\kappa (m,\tau )k(m-m^{\prime },\tau -\tau^{\prime }),}$$

where:

$$k(x,y)=\frac{1}{2\pi }\exp\left[-\frac{x^{2}}{2h_{1}}-\frac{y^{2}}{2h_{2}}\right].$$

**Figure 4.**
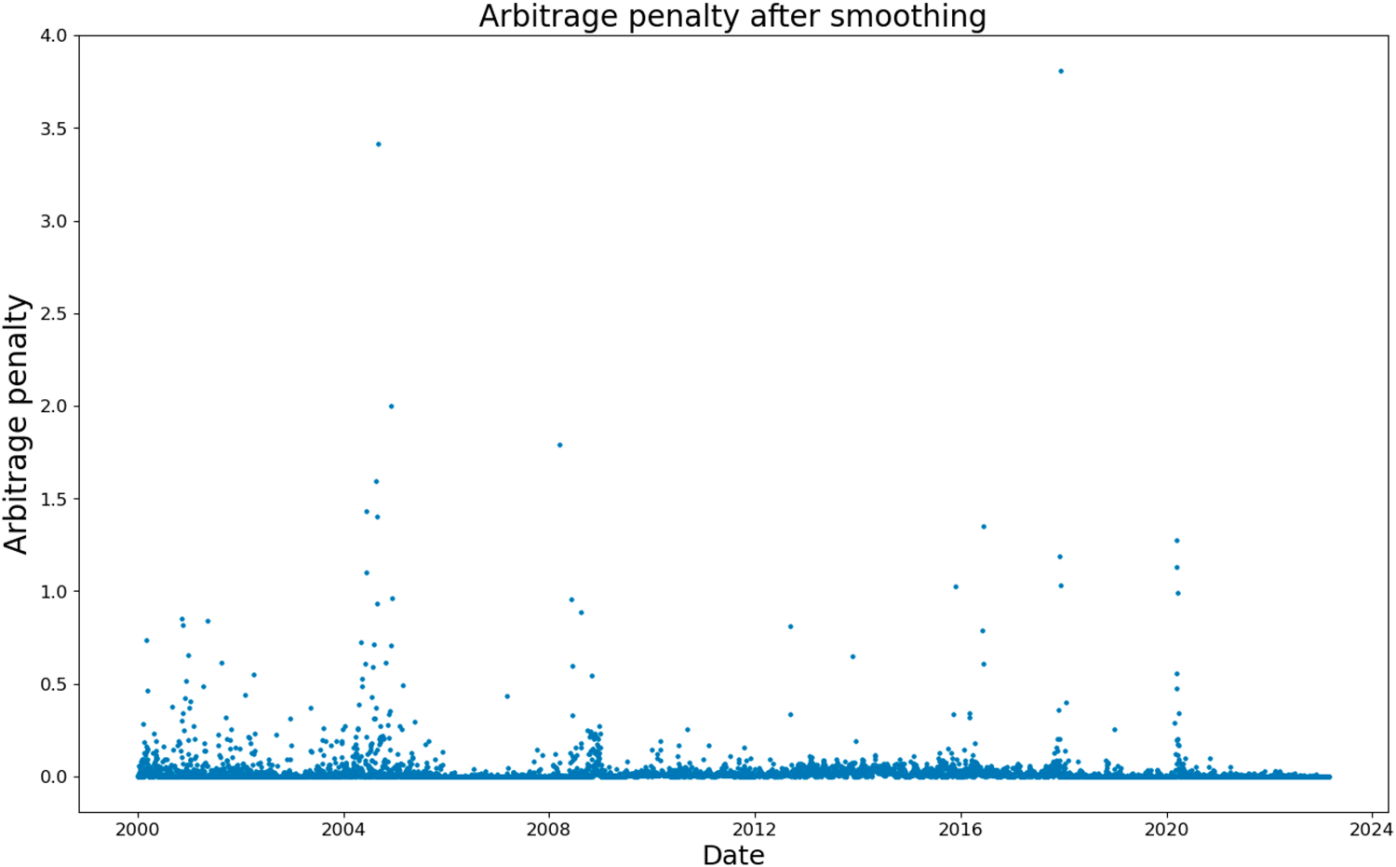
Arbitrage penalty for SPX implied volatility surface after smoothing.

In order to determine the values of the bandwidth hyperparameters $h_{1}$ and $h_{2}$, we sample a day uniformly at random from the first 100 days available (which was 31st Jan 2000) and find the pair of hyperparameters $\left(h_{1},h_{2}\right)$ minimizing the arbitrage penalty. We conduct the search over values between 0.002 and 0.1 (inclusive) in 0.002 increments, for both $h_{1}$ and $h_{2}$. The minimizer of the arbitrage penalty was the pair $\left(h_{1},h_{2})=(0.002,0.046\right)$. The resulting arbitrage penalty over the entire data set after smoothing is shown in Figure 4. Note that compared to Cont and Vuletic (2023) we include shorter times to maturity and use a different dataset.

To simplify the notation, we will use $\sigma_{t}(m,\tau )$ for the implied volatility surface obtained after smoothing, on the (m,τ) grid. For general $\sigma_{t}(m,\tau )$ we interpolate $\sigma_{t}(m,\tau )$ linearly first in moneyness, and then in time to maturity. When extrapolation is necessary, it is linear.

### 4.2. Out-of-Sample Performance

As discussed in Section 2, the main goal of an implied volatility model is to correctly capture the co-movements of implied volatilities, while satisfying static arbitrage constraints. We can measure the latter by considering the 'distance to arbitrage' using the arbitrage penalty (2). In order to measure how well VolGAN learns the dynamics and captures the co-movements of implied volatilities, we perform PCA on the generated increments, and compare them with the principal components of the data increments. Furthermore, we simulate the CBOE volatility index VIX (CBOE 2022), which is a non-linear combination of tradable calls and puts. We compare the dynamics of the simulated and market data.

#### 4.2.1. Detecting Extreme Market Events

**Figure 5.**
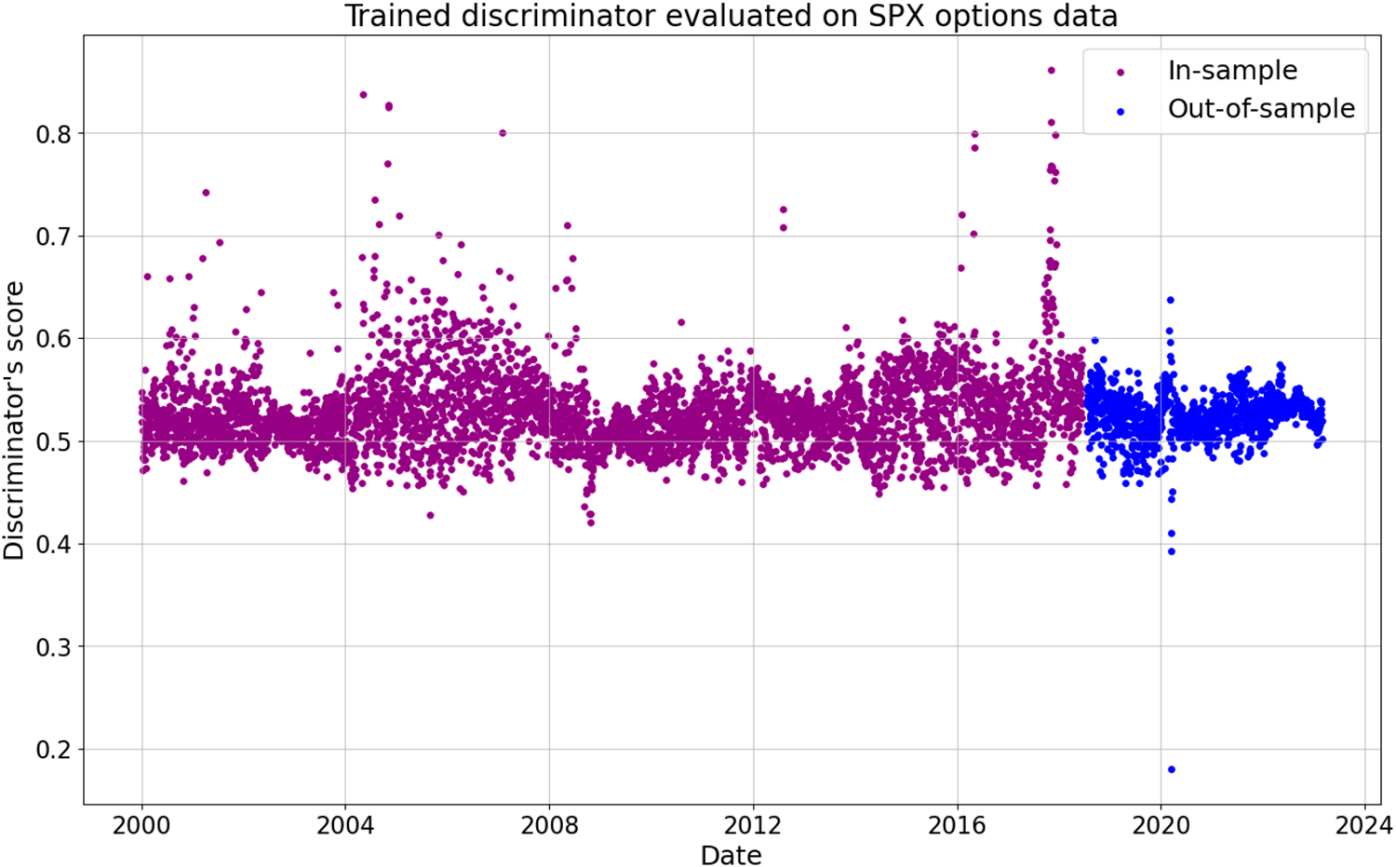
Discriminator output score on in-sample and out-of-sample SPX options data.

Firstly, we note that the trained discriminator might be used for detecting extreme market events. Figure 5 contains discriminator scores on the training and testing data. Since the discriminator has already been trained, it is of no surprise that the outputs cluster around 0.5. There are two clusters of points with scores lower than others: those corresponding to the 2008 financial crisis (in-sample) and to the start of the Covid-19 pandemic (out-of-sample). In particular, the discriminator assigns a score below 0.2 to the data from the start of the Covid-19 pandemic, highlighting the difference in this data compared to the rest of the training and test set.

#### 4.2.2. Smoothness and Arbitrage Constraints

**Figure 6.**
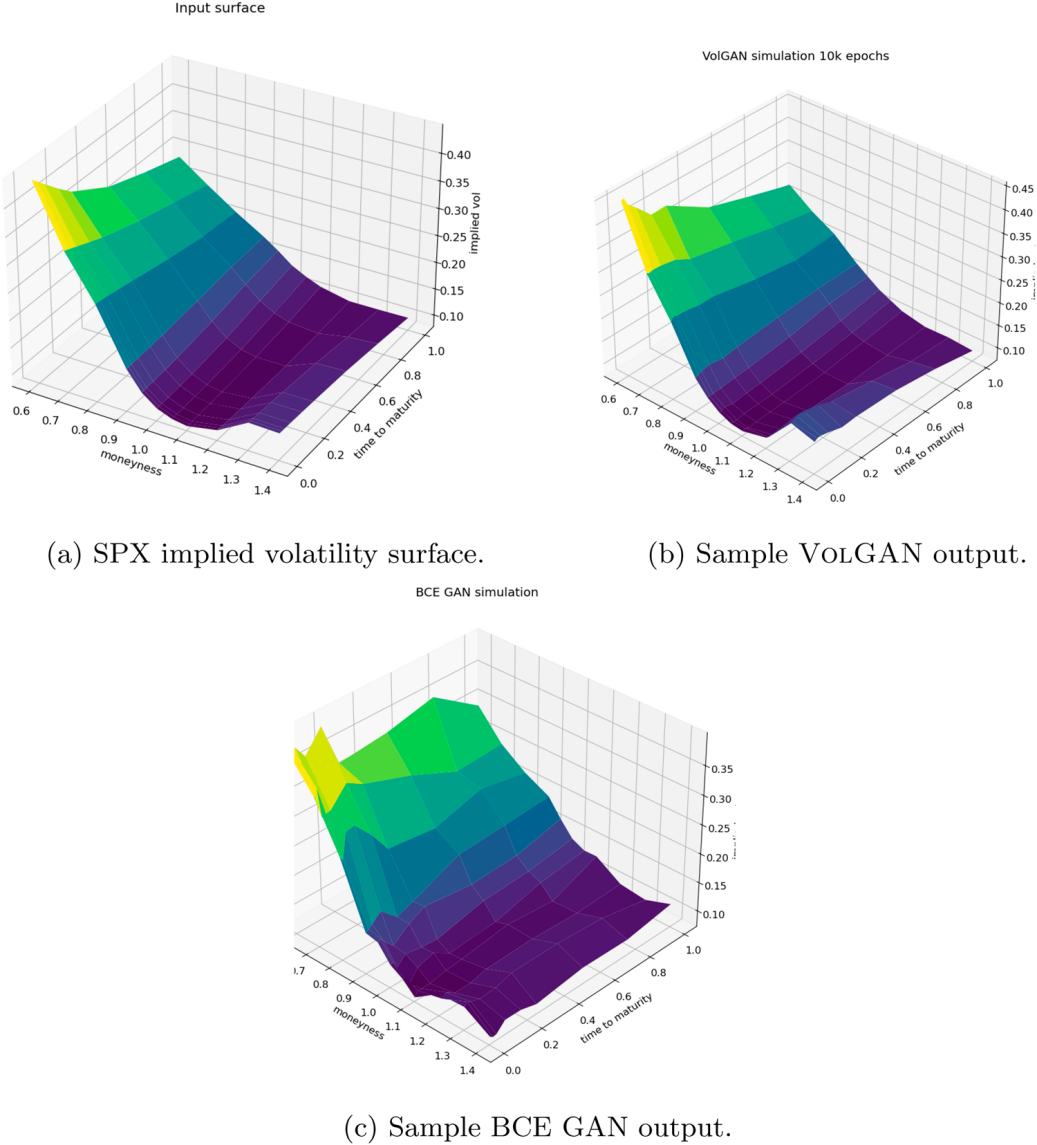
Implied volatility surfaces generated using (b) VolGAN (c) classical GAN, compared with (a) SPX implied volatility surface.

Incorporating the smoothness penalty (11)–(12) into the loss function (13) is crucial for generating smooth surfaces. As shown in Figure 6, training via the classical Binary Cross-Entropy (BCE) loss (Goodfellow et al. 2014), using the same architecture, hyperparameters, and the same number of training epochs, results in irregular surfaces.

**Figure 7.**
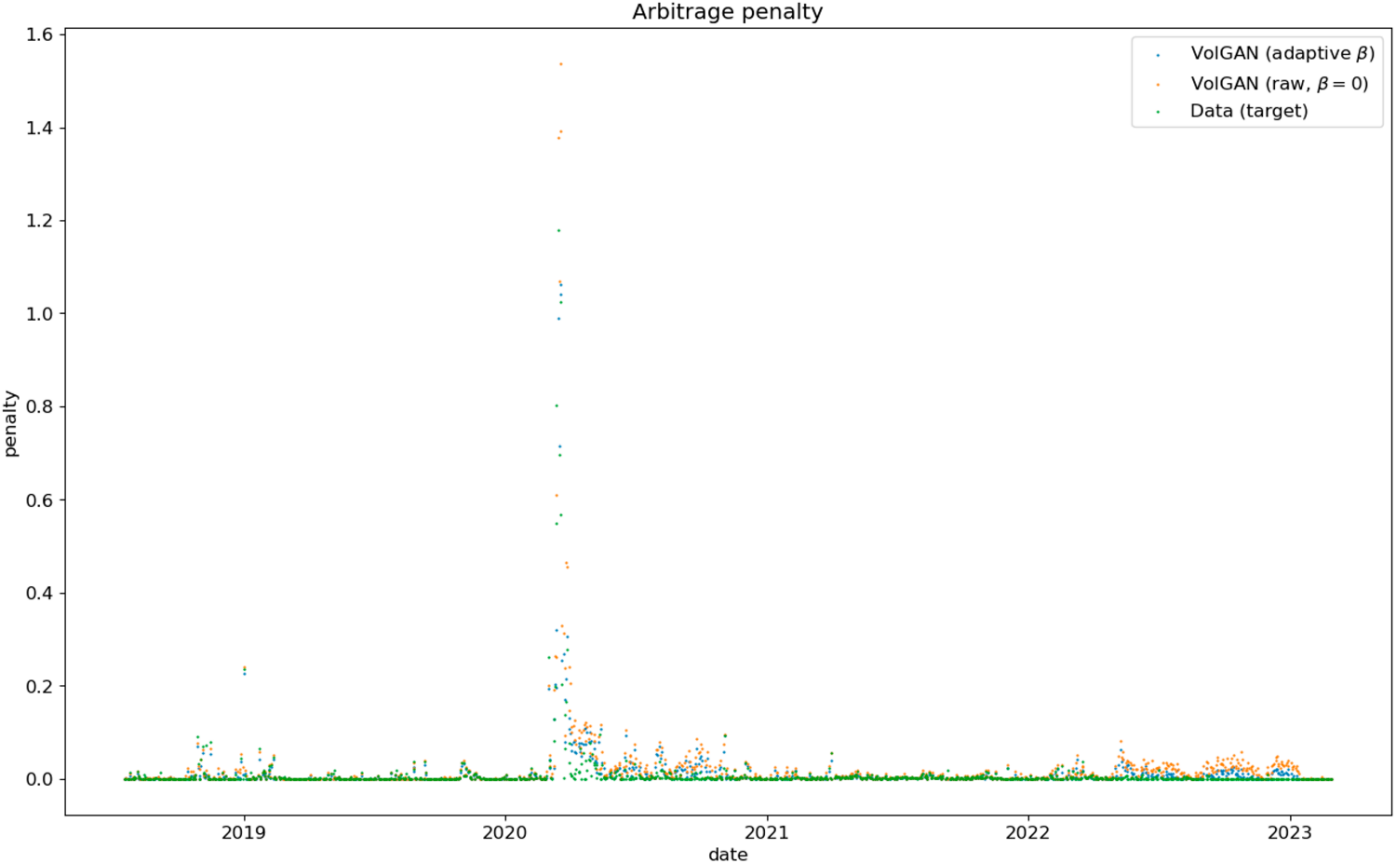
Distance to arbitrage as measured by the arbitrage penalty (2) in SPX implied volatility data (red) vs. mean arbitrage penalty of surfaces generated via VolGAN, before (blue) and after (green) scenario re-weighting.

As the input surfaces might admit static arbitrage, it is not realistic to expect outputs to be completely arbitrage-free. What is plausible, however, is for the outputs to have arbitrage penalties of the same order (or lower) than the inputs. Table 1 compares out-of-sample arbitrage penalties for SPX implied volatilities and the outputs of the BCE GAN and VolGAN with/without scenario re-weighting. Arbitrage penalties in the BCE GAN samples are observed to be high: this is linked to the previous observation that BCE GAN fails to generate smooth surfaces, resulting in failure of static arbitrage conditions which are linked to derivatives of the surfaces. In contrast, VolGAN outputs have arbitrage penalty levels similar to the input data. Scenario re-weighting leads to a low probability of selecting scenarios with static arbitrage, as shown in Figure 7, where the reduction in arbitrage is visualized. The mean, standard deviation, and median values from Table 1 correspond to the statistics of the time series displayed in Figure 7. We note that during 2022 there is more volatility in arbitrage penalty in VolGAN compared to the remainder of the test period

**Table 1.** Arbitrage penalties in SPX implied volatility market data (test set) vs generated data via GANs trained using (i) BCE loss only (ii) VolGAN loss (iii) VolGAN re-weighted scenarios (adaptive *β*). Standard deviation and median for GAN outputs correspond to the standard deviation and the median of (re-weighted) average outputs given 10,000 samples.

	Mean	Std	Median
Market data	0.0096	0.0628	0.0005
BCE GAN	2.4635	0.9086	2.3164
Raw VolGAN (before weighting)	0.0199	0.088	0.003
VolGAN (after re-weighting)	0.0127	0.0620	0.0014

#### 4.2.3. Next-Day Forecasting

We use VolGAN to generate next-day forecasts using the conditional expectation of the variable given the history, together with a 95% confidence interval obtained by considering the 2.5% and 97.5% quantiles for the following quantities of interest:

- index level $S_{t}$;
- VIX level $\sigma_{t}^{\mathrm{VIX}}$;
- a range of implied volatilities $\sigma_{t}(m,\tau )$ with
$$\tau \in \left\{\frac{1}{252},\frac{1}{52},0.25,0.125\right\},\;m\in \{0.75,1,1.25\}$$

**Figure 8.**
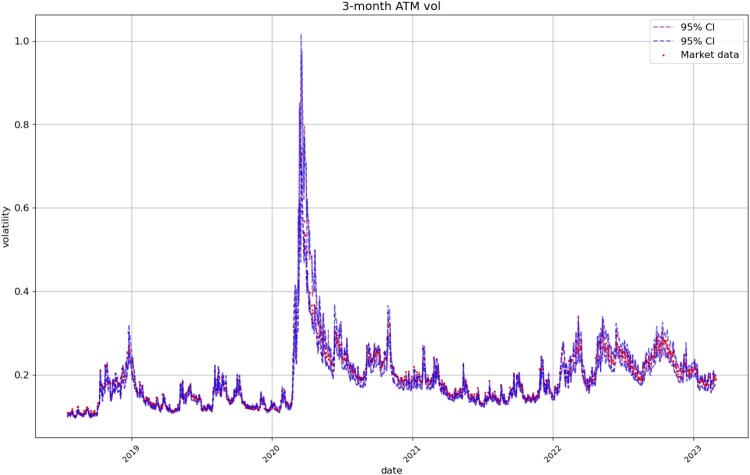
3-month ATM implied volatility ( m=1,τ=0.25): market data (red), next-day forecast $\left(E_{\text{β}}\left[\sigma_{t}(1,0.25)|a_{t-\mathrm{\Delta t}}\right]\right)$ and 95% confidence interval (blue) based on the 2.5% and 97.5% VolGAN quantiles.

**Figure 9.**
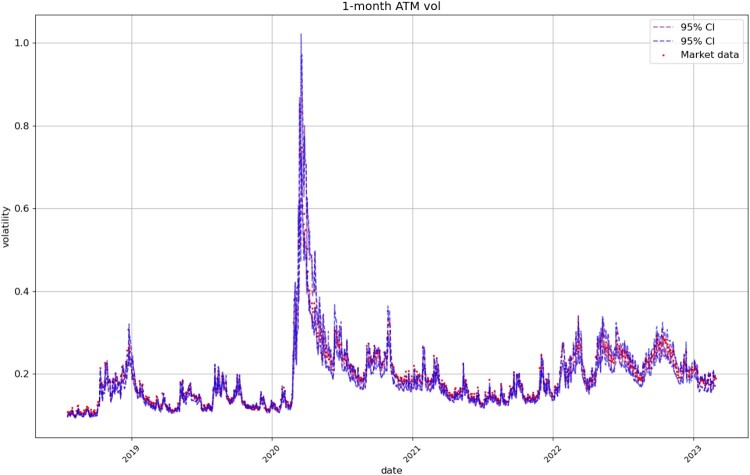
1-month ATM implied volatility ( m=1,τ=1/12): market data (red), next-day forecast $\left(E_{\text{β}}\left[\sigma_{t}(1,1/12)|a_{t-\mathrm{\Delta t}}\right]\right)$ and 95% confidence interval (blue: without re-weighting, purple: with re-weighting) based on the 2.5% and 97.5% VolGAN quantiles.

**Figure 10.**
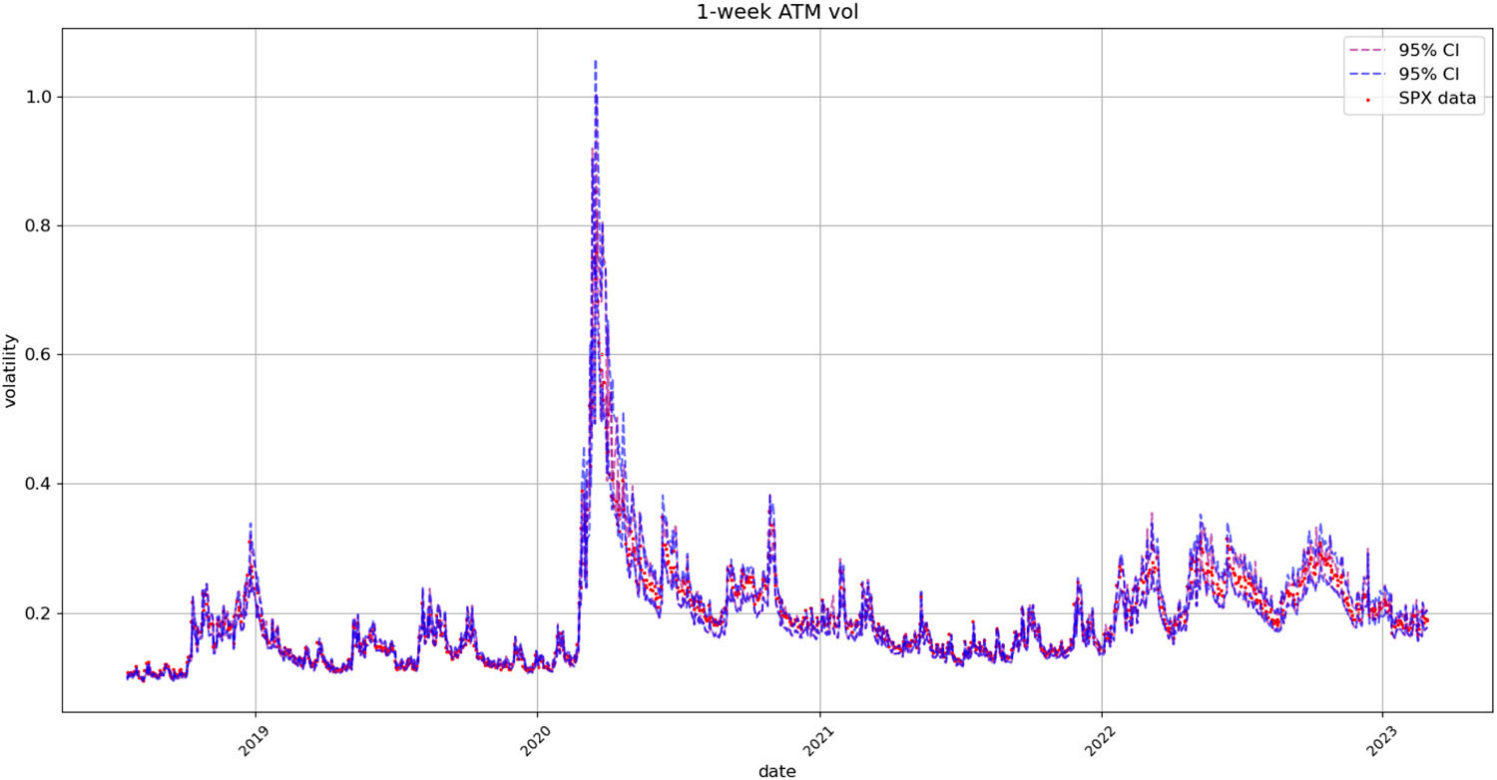
1-week ATM implied volatility ( m=1,τ=1/52): market data (red), next-day forecast $\left(E_{\text{β}}\left[\sigma_{t}(1,1/52)|a_{t-\mathrm{\Delta t}}\right]\right)$ and 95% confidence interval (blue: without re-weighting, purple: with re-weighting) based on the 2.5% and 97.5% VolGAN quantiles.

**Figure 11.**
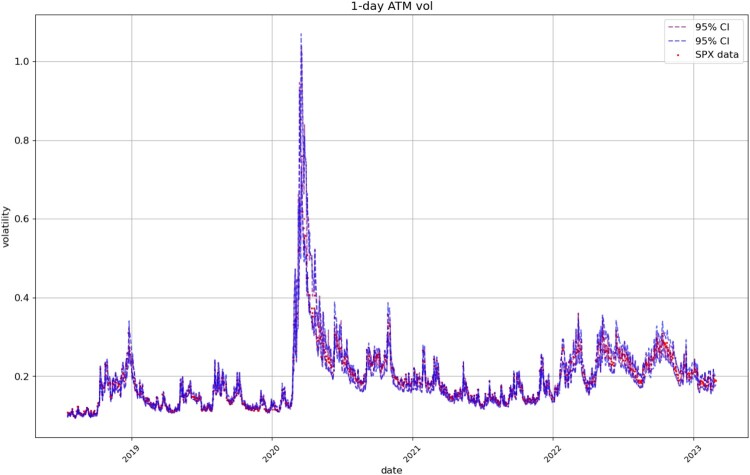
1-day ATM implied volatility ( m=1,τ=1/252): market data (red), next-day forecast $\left(E_{\text{β}}\left[\sigma_{t}(1,1/252)|a_{t-\mathrm{\Delta t}}\right]\right)$ and 95% confidence interval (blue: without re-weighting, purple: with re-weighting) based on the 2.5% and 97.5% VolGAN quantiles.

**Figure 12.**
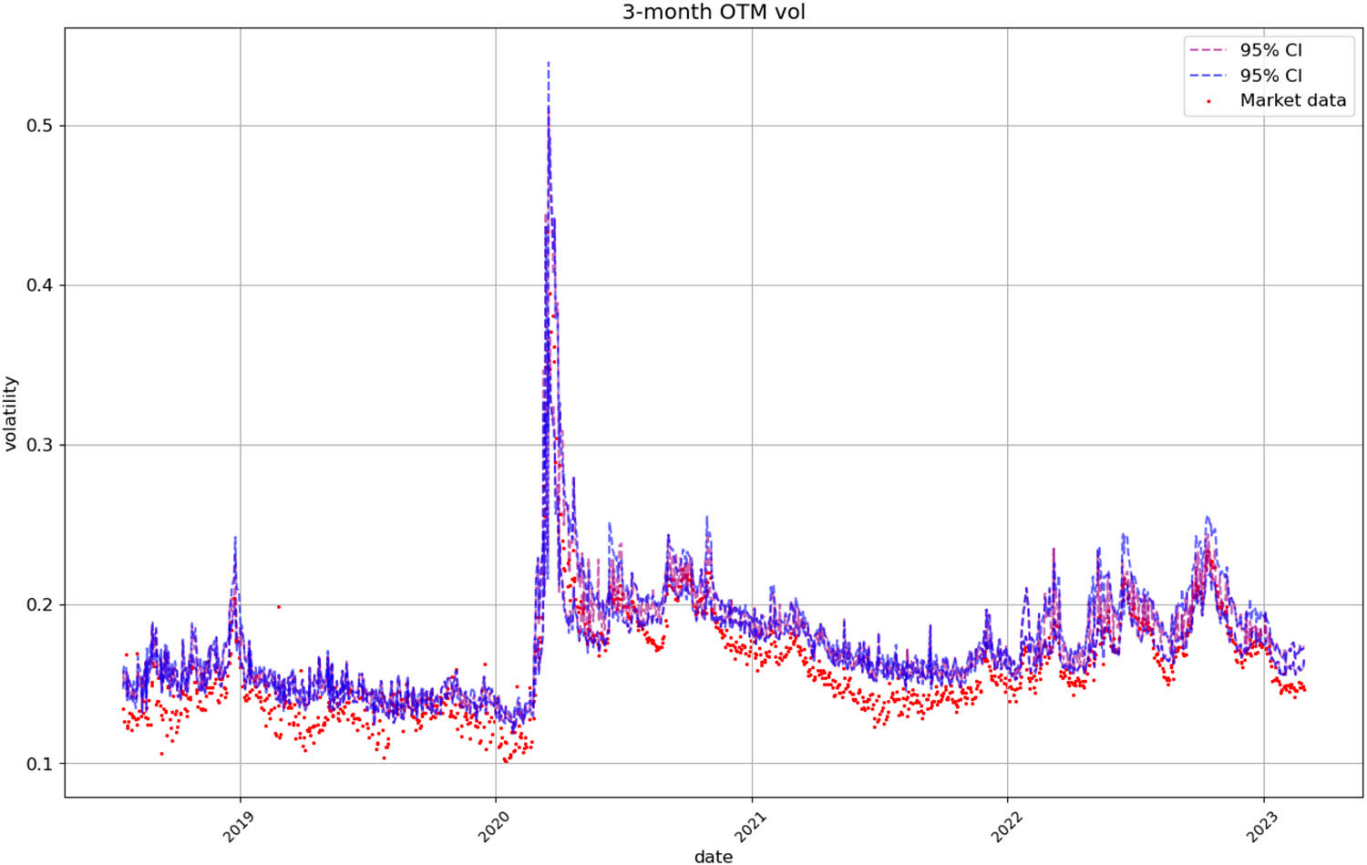
3-month OTM call implied volatility ( m=1.25,τ=0.25): market data (red), next-day forecast $\left(E_{\text{β}}\left[\sigma_{t}(1.25,0.25)|a_{t-\mathrm{\Delta t}}\right]\right)$ and the 95% confidence interval (blue: without re-weighting, purple: with re-weighting). The confidence interval is calculated based on the 2.5% and 97.5% VolGAN quantiles.

**Figure 13.**
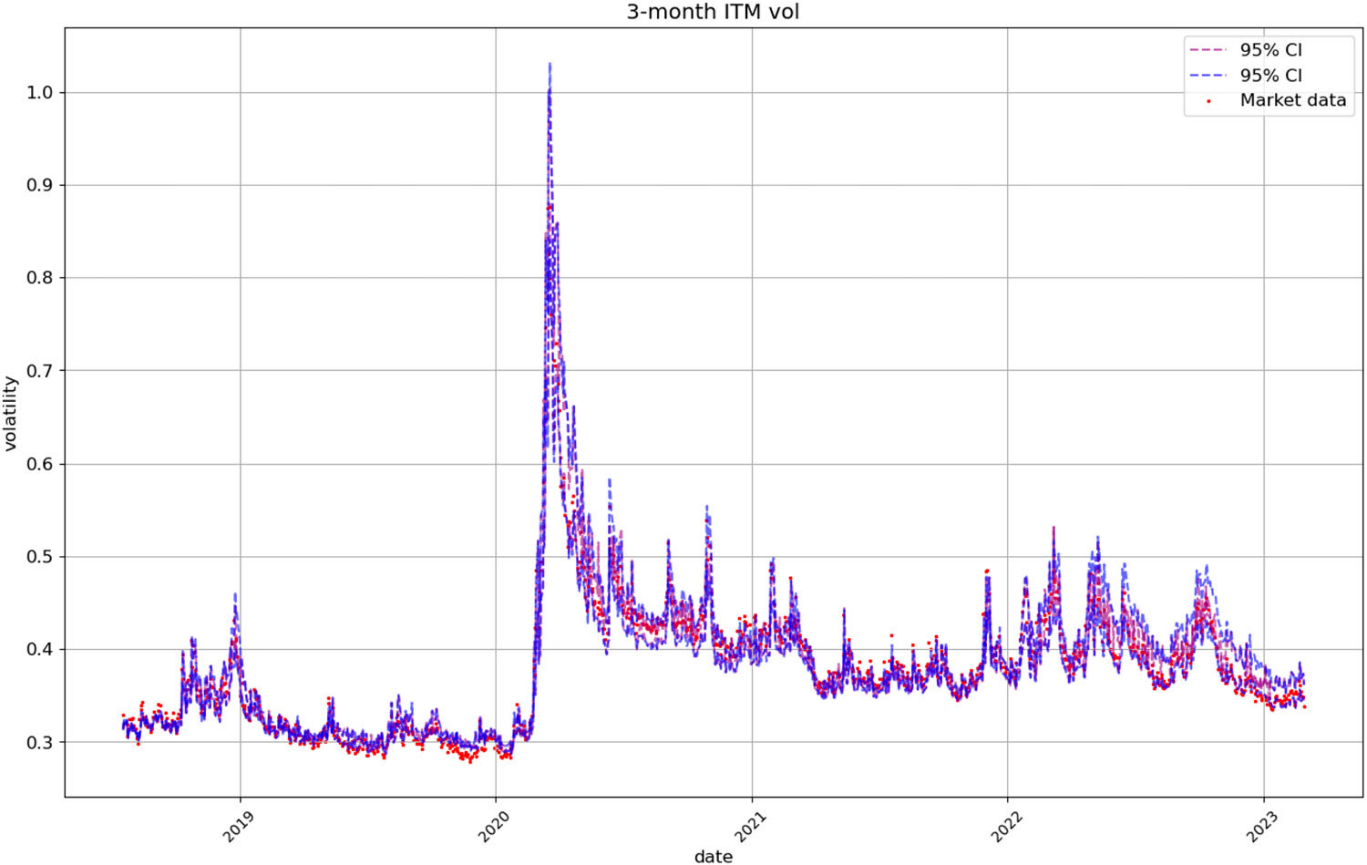
3-month ITM call implied volatility ( m=0.75,τ=0.25): market data (red), next-day forecast $\left(E_{\text{β}}\left[\sigma_{t}(0.75,0.25)|a_{t-\mathrm{\Delta t}}\right]\right)$ and 95% confidence interval (blue: without re-weighting, purple: with re-weighting). The confidence interval is calculated based on the 2.5% and 97.5% VolGAN quantiles.

Figures 8, 9, 10, 11 compare respectively the 3-month, 1-month, 1-week, and 1-day ATM implied volatility with the VolGAN one-day ahead 95% confidence interval forecast, displaying good agreement with observations. VolGAN appears to slightly overestimate implied volatility levels for *m*>1 but not for *m*<1, as shown in Figures 12 and 13.

**Figure 14.**
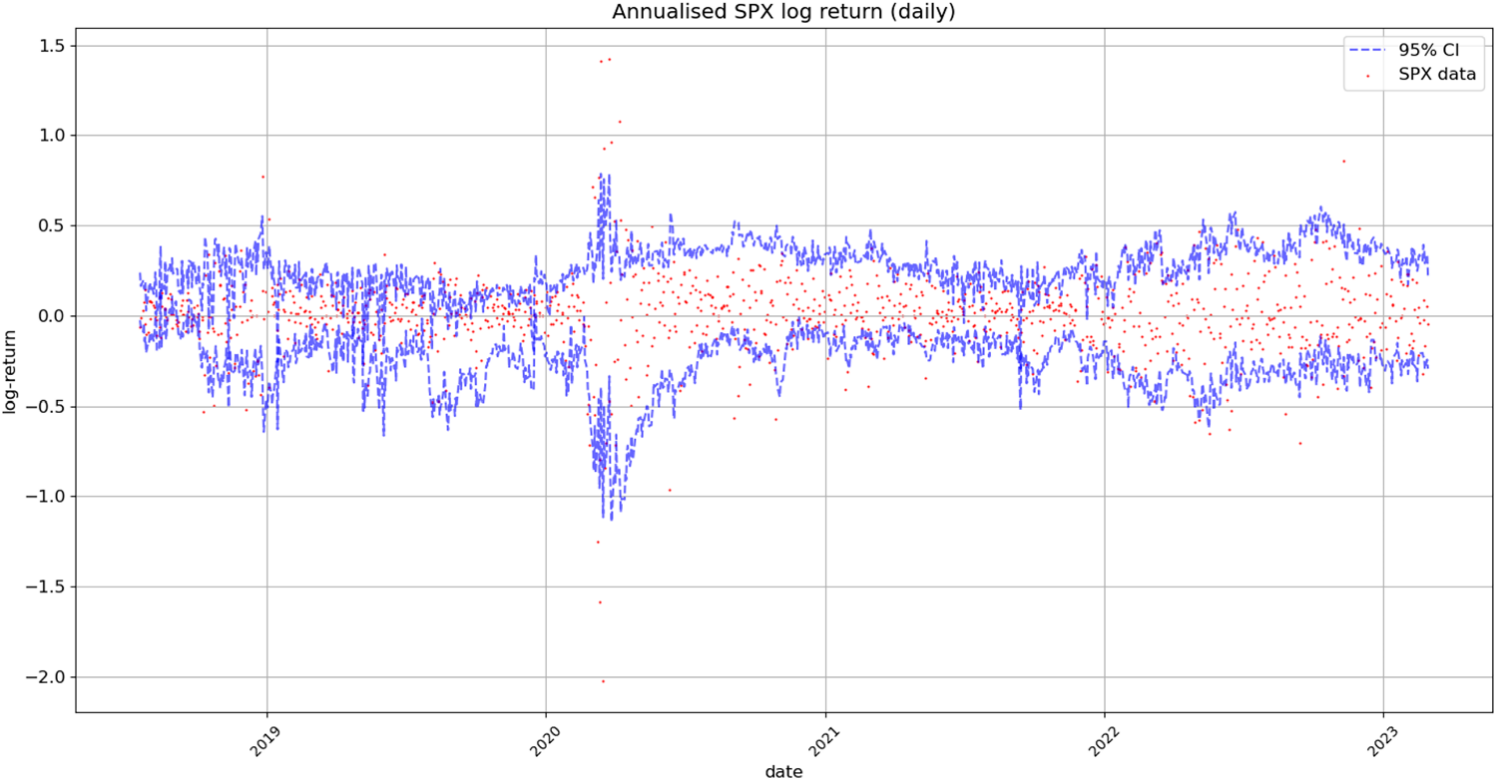
Realized and simulated SPX log-return on the test set. Market data (red), next-day forecast $\left(E_{\text{β}}\left[S_{t}|a_{t-\mathrm{\Delta t}}\right]\right)$ and the 95% confidence interval (blue: without re-weighting). The confidence interval is calculated based on the 2.5% and 97.5% VolGAN quantiles.

**Figure 15.**
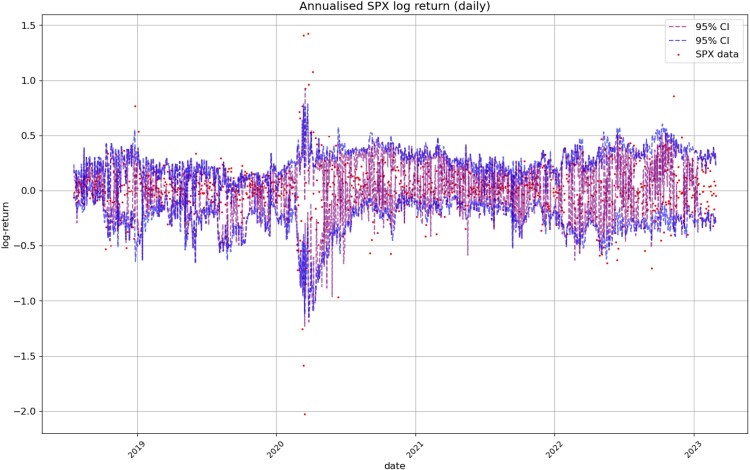
Realized and simulated SPX log-return on the test set. Market data (red), next-day forecast $\left(E_{\text{β}}\left[S_{t}|a_{t-\mathrm{\Delta t}}\right]\right)$ and the 95% confidence interval (blue: without re-weighting, purple: with re-weighting). The confidence interval is calculated based on the 2.5% and 97.5% VolGAN quantiles before and after re-weighting.

Figure 14 displays the simulated and real SPX returns, showing that VolGAN confidence intervals appropriately capture the underlying. We visualize the impact of scenario re-weighting on the confidence intervals in Figure 15. During periods of high arbitrage penalty, a small number of simulations hold most of the weight, therefore inducing very narrow confidence intervals. This behaviour is visible not just in the simulations for the underlying, but for the ATM (*m* = 1), OTM (*m* = 0.75), and ITM ( m=1.25) implied volatilities (Figures 8, 12, 13 respectively). From Figure 15, we note that if arbitrage is not penalized ( β=0), the forecasts are more accurate, including for March and April 2020. However, choosing to use the raw generator might result in static arbitrage of the mid-prices. As before, we note that the width of the confidence intervals varies with time, with the confidence intervals appearing more consistent in 2022. The raw generator ( β=0) produces stable confidence intervals for all state variables, highlighting VolGAN's stability and not requiring frequent re-calibration.

**Figure 16.**
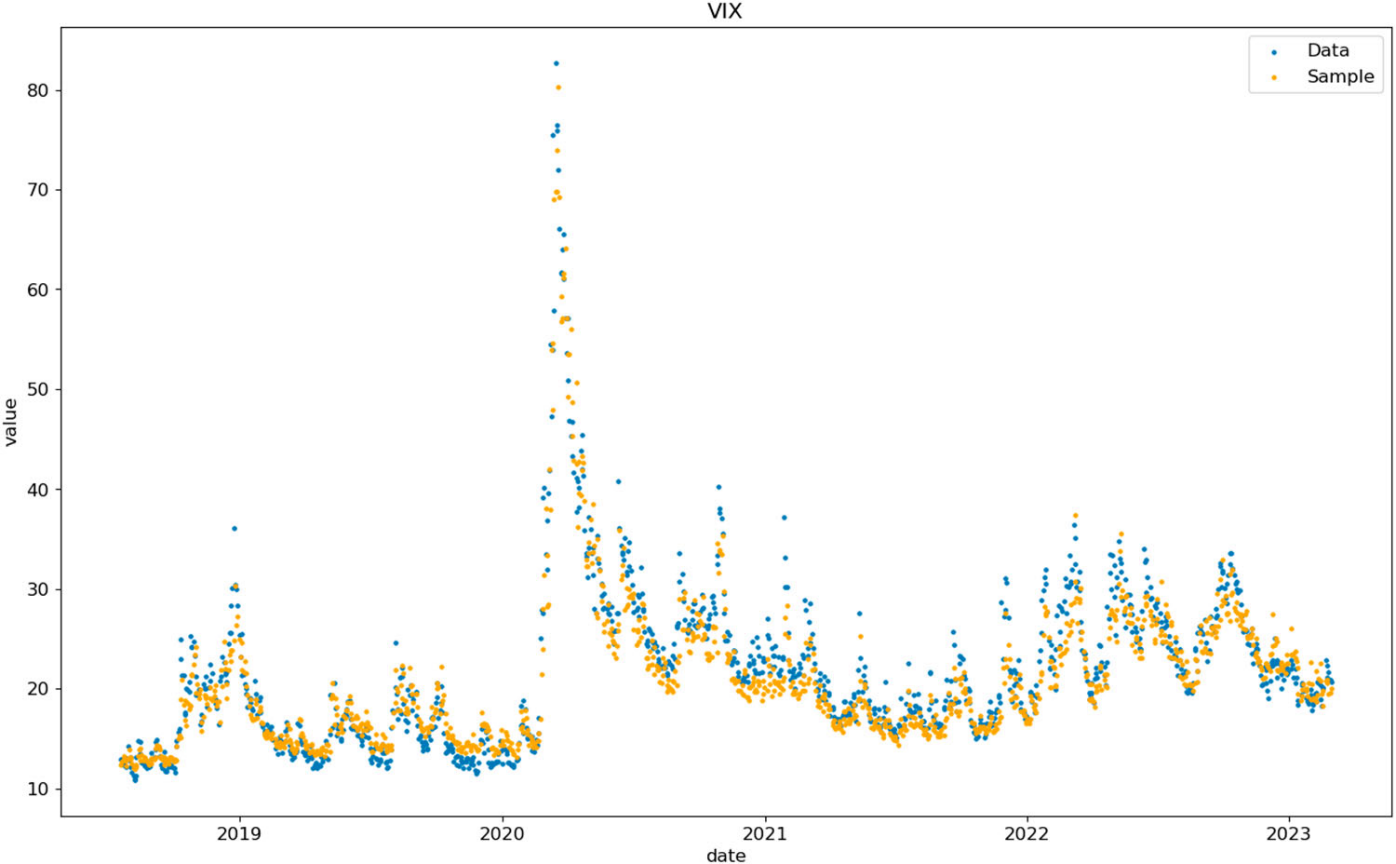
Historical vs one-day ahead simulation of VIX, on test data set.

Figure 16 compares one-day ahead simulated values of VIX, computed from its definition in terms of simulated call/put prices, with the VIX closing prices on target days in the test set. VolGAN simulations are on the same scale as VIX. Some of the differences might be coming from the discrete approximation of the log-contract used for computation of simulated VIX values (CBOE 2022).

We further investigate the prediction score in Table 2 by considering the percentage of data realizations falling below the simulated 1%, 2.5%, 97.5%, and 99% quantiles. We note that the best overall forecasts are for the underlying. VolGAN underestimates extremely high values of the implied volatility returns and VIX. Given that the volatility index is a non-linear transformation of the state variables, it is not surprising that VolGAN does not produce as stable confidence intervals as it does for the state variables. The findings from Table 2 are in line with the previous observations: VolGAN captures the state variables for which more data is available better. It is important to note that the observed behaviour is out-of-sample, four and a half years after training, including the 2020 data.

**Table 2.** Exceedance ratio for VolGAN quantiles on the test set.

Variable/Quantile	0.01	0.025	0.975	0.99
SPX return	25.32%	29.19%	82.00%	83.55%
3-month ATM vol	13.95%	15.16%	49.61%	54.61%
3-month OTM vol	76.978%	78.81%	92.85%	93.80%
3-month ITM vol	29.46%	30.32%	65.46%	69.34%
1-month ATM vol	09.82%	11.28%	42.89%	48.41%
1-week ATM vol	20.41%	22.05%	59.17%	63.22%
1-day ATM vol	19.90%	21.79%	60.12%	64.34%
VIX	34.37%	35.23%	52.67%	55.04%

As already observed in Figure 15, there are instances (of market turbulence) where *not* correcting for the presence of static arbitrage (i.e., setting β=0) actually *improves* forecasting performance. We note that when the arbitrage penalty is very low or zero, the penalization has negligible impact on the simulated confidence intervals.

Table 2 shows that chossing β=0 can in fact improves forecasts, especially for SPX returns, 1-week ATM volatility, and VIX (Table 3).

**Table 3.** Exceedance ratio for VolGAN quantiles on test set with β=0.

Variable/Quantile	0.01	0.025	0.975	0.99
SPX return	04.48%	09.39%	92.33%	93.37%
3-month ATM vol	08.52%	09.56%	64.51%	71.67%
3-month OTM vol	72.18%	73.64%	97.59%	98.02%
3-month ITM vol	20.33%	22.14%	75.62%	81.83%
1-month ATM vol	05.25%	06.55%	57.88%	66.58%
1-week ATM vol	11.80%	13.78%	72.95%	80.10%
1-day ATM vol	11.71%	13.52%	74.68%	81.65%
VIX	25.24%	25.84%	71.23%	71.18%

#### 4.2.4. Distributions and Correlations Learned by the Generator

Denote by $\rho_{t}$ the instantaneous correlation between the 1-month ATM volatility returns and the returns of the underlying at time *t*. We would like to explore whether or not VolGAN learns constant correlations. Therefore, we perform the following hypothesis test:

**
$H_{0}$:** $\rho_{t}=\rho$ is constant, **$H_{1}$:** $\rho_{t}\neq \rho$ is time-varying.

**Figure 17.**
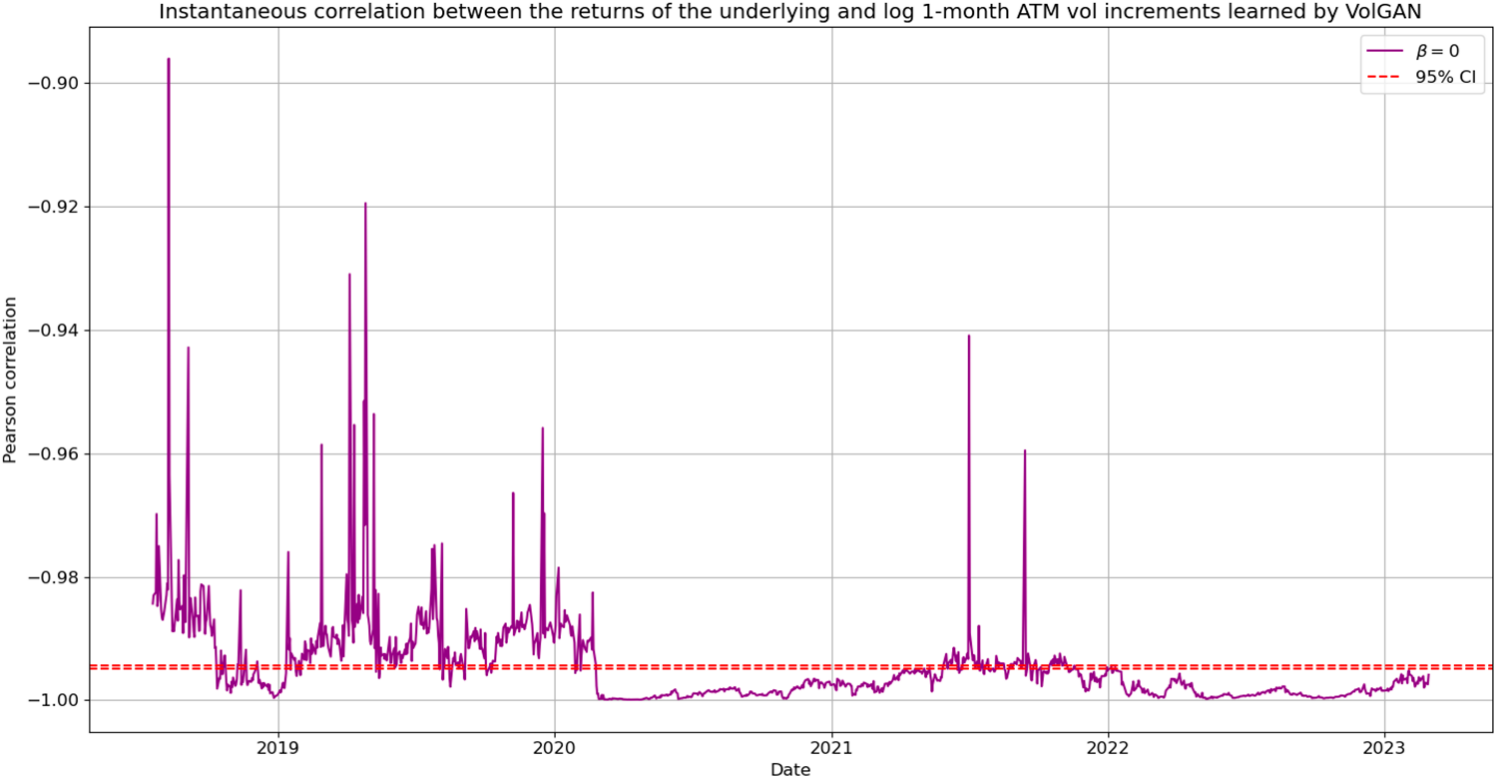
Pearson correlation between simulated index returns and 1-month ATM volatility increments (blue), with symmetric 95% confidence interval of constant correlation (red). VolGAN with β=0.

Under $H_{0}$, the 95% confidence interval for $\rho_{t}$ is given by $\left[\rho^{L},\rho^{U}\right]$, where (Bonett and Wright 2000)

$$\begin{array}{rl} \rho^{U} & =\frac{\exp(2z_{U})-1}{\exp(2z_{U})+1},\;\rho^{L}=\frac{\exp(2z_{L})-1}{\exp(2z_{L})+1};\\ z_{U} & =\frac{1}{2}\log\left[\frac{1+\rho }{1-\rho }\right]+\sqrt{\frac{1}{n-3}}z_{0.975},\;z_{L}=\frac{1}{2}\log\left[\frac{1+\rho }{1-\rho }\right]-\sqrt{\frac{1}{n-3}}z_{0.975}, \end{array}$$

where *n* is sample size. Estimating *ρ* by the sample mean of $\rho_{t}$ on the test set, in Figure 17 we plot $\rho_{t}$ and the 95% confidence interval $\left[\rho^{L},\rho^{U}\right]$. We note that $\rho_{t}$ is away from the confidence interval of $H_{0}$, indicating strong evidence against $H_{0}$. VolGAN learns time-varying instantaneous correlations which would be difficult to capture with a parametric model.

**Figure 18.**
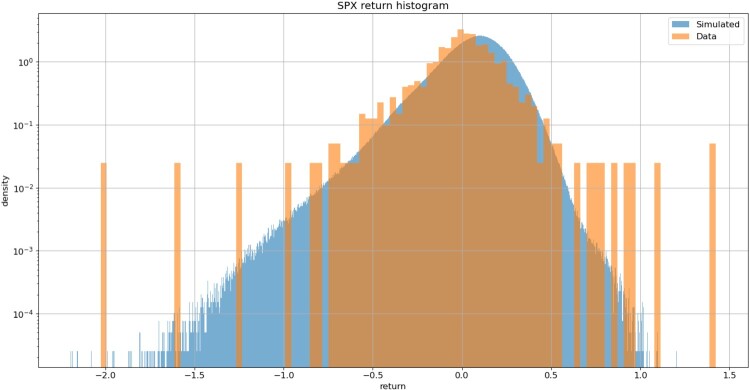
Simulated index returns (blue) exhibit asymmmetric, exponentially decaying tails. VolGAN with β=0.

**Figure 19.**
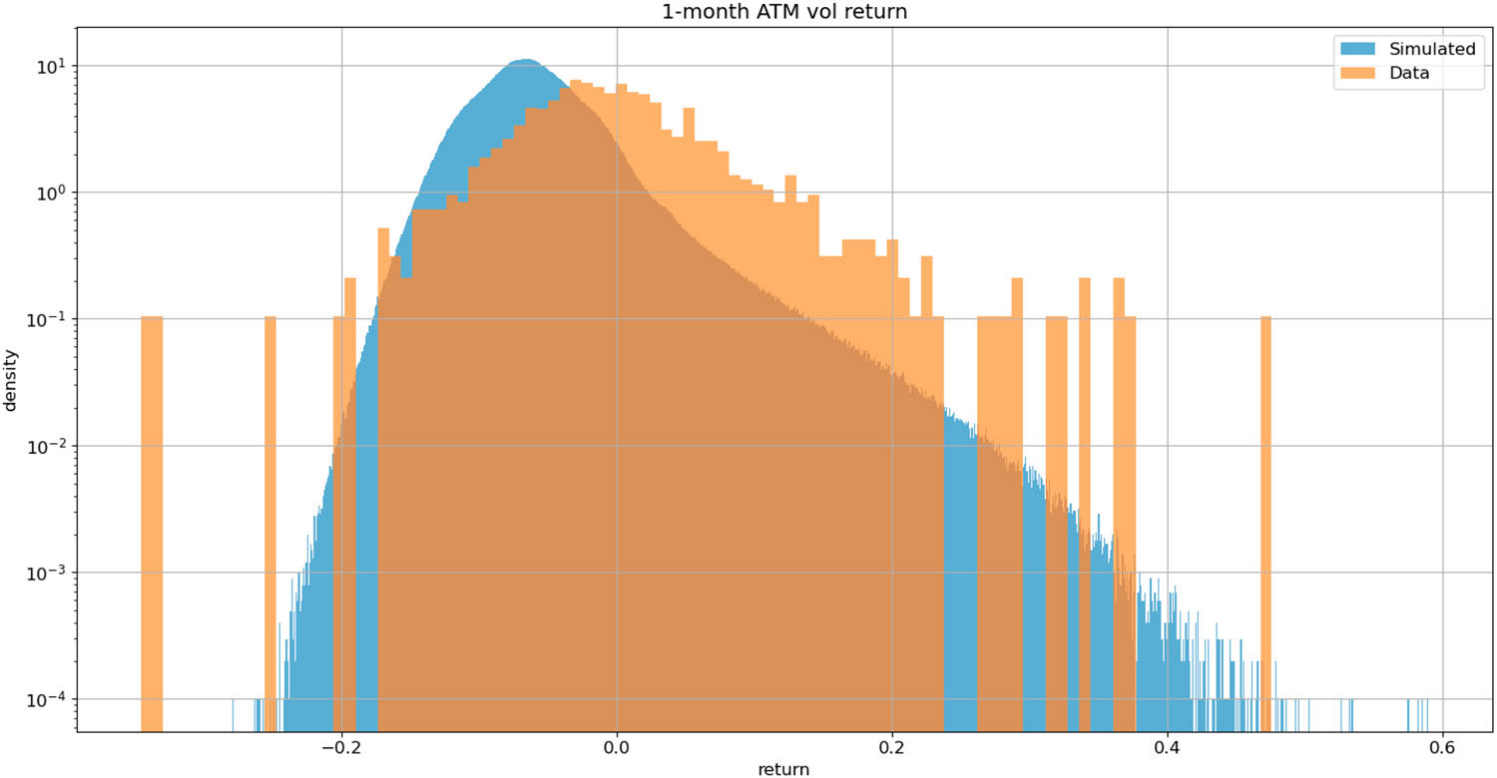
Simulated 1-month ATM volatility increments (blue) exhibit asymmmetric, exponentially decaying tails. VolGAN with β=0.

We compare the (simulated) distributions of the daily returns for the underlying and 1-month ATM volatility with the corresponding empirical distributions and with Gaussian distributions with the same mean and variance. Figures 18 and 19 shows that simulated index returns and ATM volatility increments have asymmetric, non-Gaussian and exponentially decaying tails. Such non-Gaussian, asymmetric distributions are difficult to capture in a model with Brownian increments.

#### 4.2.5. Principal Component Analysis

In order to investigate VolGAN's ability to appropriately capture the implied volatility co-movements, we perform out-of-sample principal component analysis on the simulated log increments of implied volatility. We compare the first three simulated principal components with the corresponding PCs of the data realizations. When performing PCA on four and a half years of SPX implied volatility data, the eigenvectors change depending on the period of observation, but nonetheless correspond to *level, skew* and *curvature*. In Table 4 we show variance explained by the first three eigenvectors in the testing data and in the VolGAN simulations. The significance of the first two principal components is very similar in the test data and in VolGAN. The third principal component is more significant in the simulated data compared to the market data.

**Table 4.** Out-of-sample (two years after training) percentage of variance explained by the top three principal components of the simulated and the data log implied volatility increments. The VolGAN column contains the average ±1.96× standard deviation of the observed values, across 1000 VolGAN samples.

Rank	Data	VolGAN
First	51.25%	45.31±1.84%
Second	34.00%	25.69±0.88%
Third	05.01%	12.76±0.55%

**Figure 20.**
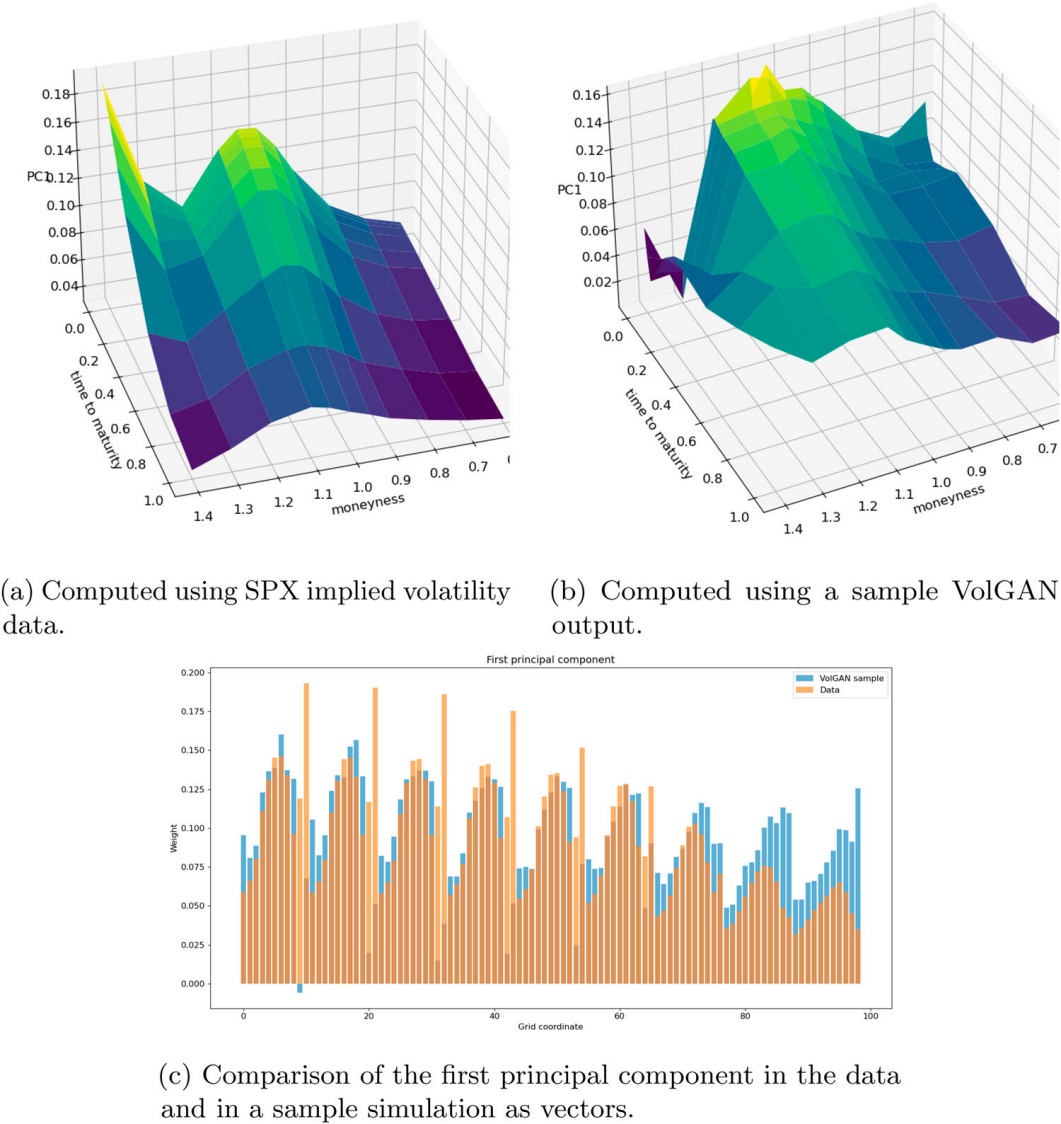
Out-of-sample (four years after training) first principal component of the daily log implied volatility increments. (a) Computed using SPX implied volatility data. (b) Computed using a sample VolGAN output and (c) Comparison of the first principal component in the data and in a sample simulation as vectors.

**Figure 21.**
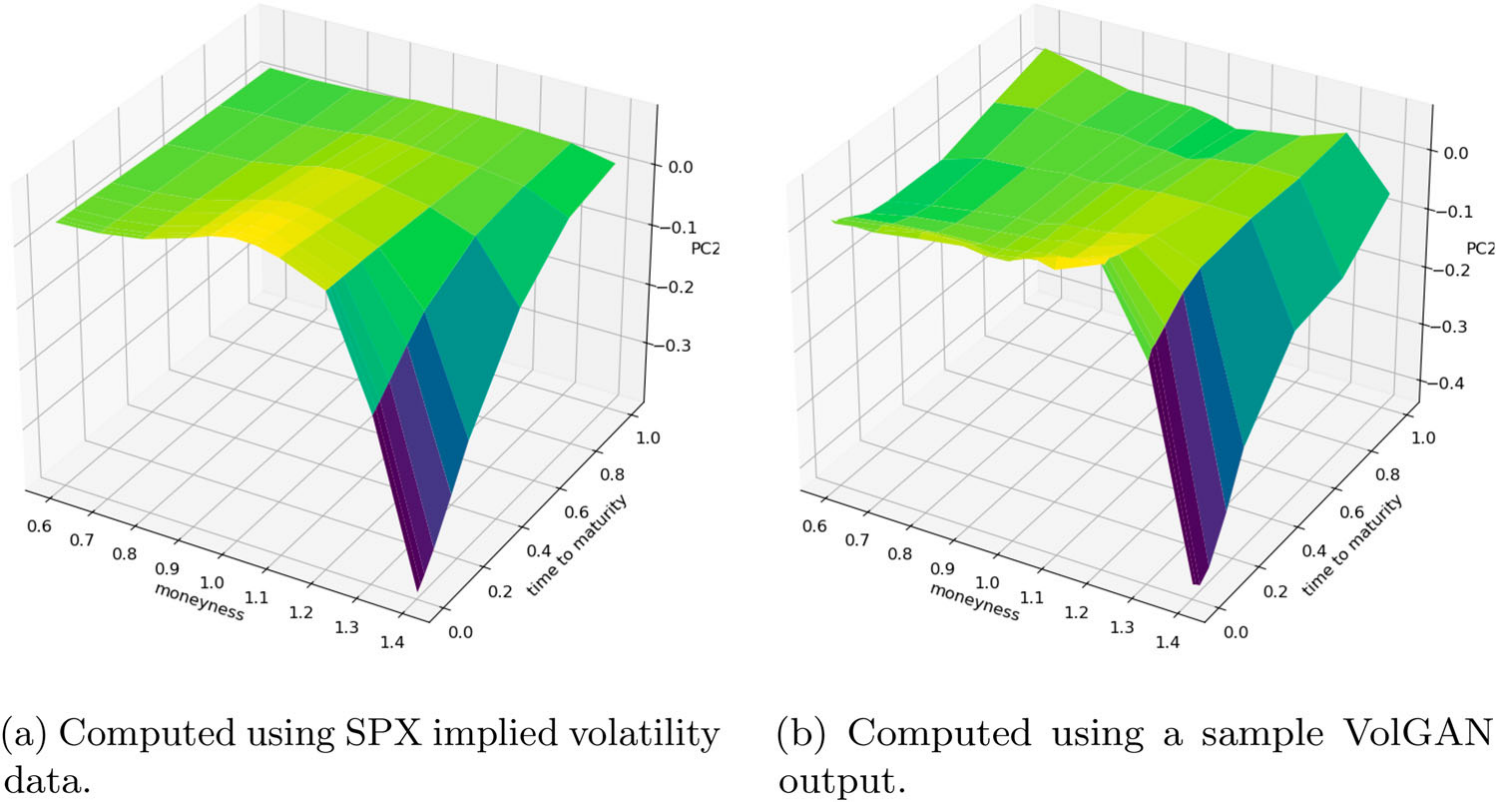
Out-of-sample (four years after training) second principal component of the daily log implied volatility increments. (a) Computed using SPX implied volatility data and (b) Computed using a sample VolGAN output.

**Figure 22.**
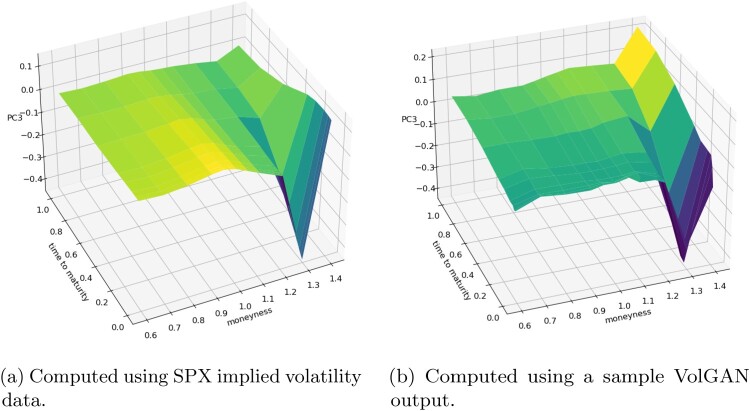
Out-of-sample (four years after training) third principal component of the daily log implied volatility increments. (a) Computed using SPX implied volatility data and (b) Computed using a sample VolGAN output.

The first principal components of the sample VolGAN implied volatility log-returns and of the corresponding SPX implied volatility market data are displayed in Figure 20. Both surfaces are consistently positive, indicating that they might have a *level* interpretation. The second eigenvectors of both SPX implied volatility market data and of the simulated scenarios (Figure 21) can be interpreted as *skew*, while the third eigenvectors (Figure 22) can be interpreted as *curvature*. Figures 20, 21, 22 reflect on the clear resemblance between the principal components of the SPX implied volatility market data and of the VolGAN simulations, showing that VolGAN is able to dynamically learn the covariance structure of implied volatility co-movements.

In order to quantify the similarity between the PCs of the simulated and the market data, we calculate the inner product between them (as vectors) over 1000 i.i.d. VolGAN samples. A value of one would indicate perfect alignment of the eigenvectors. From Table 5 we note that the first two inner products (PC1 with PC1, and PC2 with PC2) are very close to one, especially considering that the quantities are for the out-of-sample data. The inner product between the third eigenvectors of simulations and data realizations is lower than for the first two PCs, but it is nevertheless high. Furthermore, there is close resemblance in the physical interpretations of the third eigenvectors. Therefore, VolGAN is able to learn the most important eigenvectors both qualitatively and quantitatively, showing the ability to learn the covariance structure of the SPX implied volatility co-movements.

**Table 5.** Out-of-sample inner products of eigenvectors of the covariance matrices of daily log-returns of SPX implied volatility and the corresponding eigenvectors of the covariance matrix of VolGAN implied volatility increments.

Rank	Mean	Median	Standard deviation
First	0.921	0.922	0.009
Second	0.921	0.922	0.011
Third	0.798	0.798	0.011

#### 4.2.6. Correlation Structure of Variables

We further investigate VolGAN's ability to simulate realistic scenarios by examining how well it reproduces correlations between variables of interest. First, we consider the relationship between the projections of the log-implied volatility increments onto the first three principal components and the log-returns of the underlying.

Table 6 considers the correlations between index returns and the projections of the log-implied volatility increments onto the first three principal components, comparing their values in SPX options data with those in VolGAN scenarios. The correlation between the first projection process and the simulated log-returns of the underlying is close to that of market data, whereas the projections on the second and the third principal component have slightly stronger correlations with the returns of the underlying in VolGAN than they do in the SPX implied volatility market data. Nevertheless, both quantities are on the same scale. The correlation between the projection on the third principal component and the underlying is low both in VolGAN and in the options data.VolGAN is able to reproduce the correct relationships between the projection processes and the returns of the underlying: the correlations between the returns of the underlying and the projections of the log implied volatility increments onto the level and skew principal component are negative, whereas the correlation with the projection onto the curvature principal component is low (and positive).

**Table 6.** Pearson correlation between (simulated) SPX log-returns and the projections of the (simulated) log-implied volatility increments on the principal components. The VolGAN column contains the mean ±1.96× standard deviation of the observed Pearson correlations across 1000 samples. Implied volatility increments in the *Data (train)* column are projected onto the principal components of the test data for consistency.

PC rank	Data (test)	VolGAN (test)	Data (train)
First	−0.76	−0.84±0.024	−0.34
Second	−0.29	−0.38±0.055	−0.32
Third	0.06	−0.16±0.020	0.28

In order to correctly capture joint dynamics of implied volatilities and the underlying index, we are interested in the relationship between the log increments of the index ( $\mathrm{\Delta log}S_{t}$), the projection of the log-implied volatility increments onto the first principal component ( $\Delta X_{t}^{1}$), the log increments of the 1-month at-the-money implied volatility ( $\mathrm{\Delta log}\sigma_{t}^{\mathrm{ATM}}$), and the log increments of VIX ( $\mathrm{\Delta log}v_{t}$). Table 7 contains average Pearson correlations for VolGAN simulations (blue) vs the market data (red) on the test set. VolGAN simulations exhibit similar correlations between all variables of interest. The correlations between the VIX increments and the increments of the other state variables are slightly lower in VolGAN scenarios compared to the data observation on the test set. However, they are of the correct sign and magnitude. The correlation between $\mathrm{\Delta log}S_{t}$ and $\mathrm{\Delta log}\sigma_{t}^{\mathrm{ATM}}$ became significantly higher in magnitude in the period used for testing compared to the period used for training, as noted in Cont and Vuletic (2023), which could explain why VolGAN results in slightly stronger correlations between the the index returns and $\Delta X_{t}^{1}$, that is $\mathrm{\Delta log}\sigma_{t}^{\mathrm{ATM}}$.

**Table 7.** Out-of-sample (4.5 years after training including Covid) average Pearson correlation for simulated vs real values of log-returns of SPX ( $\mathrm{\Delta log}S_{t}$), implied volatility level factor ( $\Delta X_{t}^{1}$), 1-month ATM volatility ( $\mathrm{\Delta log}\sigma_{t}^{\mathrm{ATM}}$) and VIX ( $\mathrm{\Delta log}v_{t}$). Average VolGAN outcome (blue) and data (red).

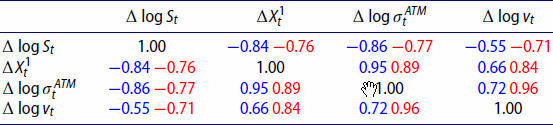

We repeat the analysis for the first year in the test set in Table 8. We observe that the magnitude of the correlation between the log-increments of VIX and the log SPX returns is a bit lower in simulations compared to the data. In the last year of the test set (Feb 2022-Feb 2023), the correlations between the simulated values of log SPX returns, increments of the level factor, and the at-the-money vol returns increase in magnitude, as noted in Table 9. We observe that the same is true for the actual values stemming from the data. The correlation structure of the simulated variables is consistent with the market, regardless of the testing period.

**Table 8.** First year out-of-sample average Pearson correlation for simulated vs real values of log-returns of SPX ( $\mathrm{\Delta log}S_{t}$), implied volatility level factor ( $\Delta X_{t}^{1}$), 1-month ATM volatility ( $\mathrm{\Delta log}\sigma_{t}^{\mathrm{ATM}}$) and VIX ( $\mathrm{\Delta log}v_{t}$). Average VolGAN outcome (blue) and data (red).

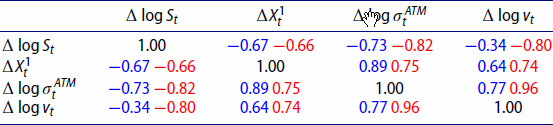

**Table 9.** Last year out-of-sample average Pearson correlation for simulated vs real values of log-returns of SPX ( $\mathrm{\Delta log}S_{t}$), implied volatility level factor ( $\Delta X_{t}^{1}$), 1-month ATM volatility ( $\mathrm{\Delta log}\sigma_{t}^{\mathrm{ATM}}$) and VIX ( $\mathrm{\Delta log}v_{t}$). Average VolGAN outcome (blue) and data (red).

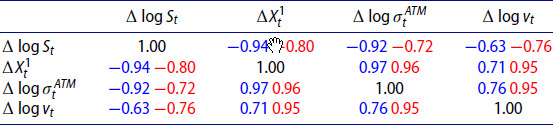

Our results demonstrate that VolGAN is able to simulate realistic co-movements for implied volatilities across a range of moneyness and maturities, as well as the underlying index and VIX: in particular we are able to reproduce time-varying correlations between increments of these variables.

## 5. Application to Hedging and Risk Management of Option Portfolios

The main motivation for generative models in finance is their use for risk management and hedging. We will now examine how VolGAN may be used to design effective hedging strategies for options portfolios. In contrast with model-based approaches such as Deep hedging (Buehler et al. 2019), our approach is completely *data-driven* and model-free, in the spirit of Hutchinson, Lo, and Poggio (1994).

Consider a portfolio whose value $V_{t}=V(t,S_{t},\sigma_{t})$ is determined by the price of the underlying asset $S_{t}$ and the implied volatility surface $\sigma_{t}(.,.)$. This may be a portfolio of call/put options, or any portfolio of derivatives which may be priced by calibrating a pricing model to the market volatility surface $\sigma_{t}$.

As an example, we will focus below on the case where the target portfolio is composed of (possibly illiquid) call or put options options, with the same expiry date *T*.

A typical problem is to hedge this portfolio with other, more liquid call/put options and the underlying. Let H be the set of hedging instruments. A hedging strategy will be a self-financing portfolio composed of instruments i∈H. If $\phi_{t}^{i}$ is the position (hedge ratio) in a hedging instrument, the value of the hedging portfolio $\hat{V}$ satisfies

(22) $$\Delta {\hat{V}}_{t}={\hat{V}}_{t+\mathrm{\Delta t}}-{\hat{V}}_{t}=\sum_{i\in H}\phi_{t}^{i}\Delta H_{t}^{i}+r_{t}\left({\hat{V}}_{t}-\sum_{i\in H}\phi_{t}^{i}H_{t}^{i}\right)\mathrm{\Delta t},$$

where Δt is the hedging frequency and $r_{t}$ is the risk-free interest rate. Set

(23) $${\hat{V}}_{0}=V_{0}.$$

and denote by $Z_{t}$ the tracking error, which is also the PnL of the hedged position:

(24) $$Z_{t}=V_{t}-{\hat{V}}_{t}.$$

We will now compare several methods for choosing the hedging instruments and hedge ratios.

### 5.1. Delta Hedging

Here the only hedging instrument is the underlying $H_{t}^{0}=S_{t}$ and the hedge ratio is set to be the overall (Black–Scholes) delta of the portfolio:

(25) $$\phi_{t}^{0}=\Delta_{t}^{V}=\frac{\partial V}{\partial S}(t,S_{t},\sigma_{t}).$$

### 5.2. Delta-Vega Hedging

This method achieves vega-neutrality by computing a sensitivity $\kappa_{t}^{V}$ to a shift in implied volatilities (e.g., a parallel shift) and hedging against this move by including an option in the hedging set. As before, let $H_{t}^{0}$ be the underlying and let $H_{t}^{1}$ be the option used as a hedging instrument. Typically this is a liquid call or put option. Denoting by $\kappa_{t}^{H}$ the vega of the option used as hedging portfolio, we achieve vega-neutrality by choosing

(26) $$\phi_{t}^{1}=\frac{\kappa_{t}^{V}}{\kappa_{t}^{H}},\;\phi_{t}^{0}=\Delta_{t}^{V}-\phi_{t}^{1}\Delta_{t}^{H}.$$

where $\Delta_{t}^{H}$ is the delta of the option used for hedging.

### 5.3. Scenario-Based Regression Hedging

We now explain how to use VolGAN to design a completely data-driven hedging strategy.

Given a set of hedging instruments $H^{i},i\in H$ and a set of VolGAN next-day scenarios $\left\{\omega_{j},j=1\ldots N\right\}$, we determine the hedge ratios $\phi_{t}^{i}$ by interpreting the one-step evolution of the portfolio

$$V_{t+\mathrm{\Delta t}}-V_{t}=\sum_{i\in H}\phi_{t}^{i}\left(H_{t+\mathrm{\Delta t}}^{i}-H_{t}^{i}\right)+(Z_{t}-Z_{t+\mathrm{\Delta t}}),$$

as a regression equation across VolGAN scenarios:

(27) $$\Delta V_{t}=V_{t+\mathrm{\Delta t}}(\omega_{j})-V_{t}=A_{t}+\sum_{i\in H}\phi_{t}^{i}\left(H^{i}(\omega_{j}{)}_{t+\mathrm{\Delta t}}-H_{t}^{i}\right)+\epsilon_{j}.$$

Therefore, the hedge ratios $\phi_{t}^{i}$ can be obtained by regressing the simulated values of $\Delta V_{t}$ on the corresponding simulated values of $\left\{\Delta H_{t}^{i}\right\}$.

### 5.4. Choice of Hedging Instruments

Delta-vega hedging rules provide no insight on the choice of the hedging instrument and can be achieved in principle using any option used as a hedging instrument. It is common to use ATM calls, but vega is sensitive to moves in the underlying asset. Our regression approach allows choosing the hedging instruments from a larger set of potential candidates $H_{0}$ using variable selection methods such as LASSO, which induces as sparsity and stability.

### 5.5. Example: Hedging a Straddle

In order to test how well VolGAN captures the joint dynamics of the implied volatility surface and the underlying index, we perform a hedging exercise where the portfolio consists of a one-month call and put option with strike $K=1.2S_{0}$. We will compare the following:

- **BS delta-vega hedge**: Black–Scholes delta-vega hedge using a call option initiated ATM at t=0.
- **BS delta hedge**: Black–Scholes delta hedging.
- **VolGAN + LASSO**: VolGAN daily regression hedge with multiple options selected via LASSO regression, without scenario re-weighting ( β=0).
- **VolGAN + ATM**: VolGAN daily regression hedge using a call option initiated ATM at t= 0, without scenario re-weighting ( β=0).
- **VolGAN + LASSO + Scenario Weighting**: VolGAN daily regression hedge using call initiated ATM at t= 0, with scenario re-weighting.

The extended hedging set $H_{0}$ used for LASSO selection in data-driven hedging via VolGAN consists of calls and puts with the same expiry as the straddle position (one month from the start) and strikes of:

-
$0.9S_{0}$, $0.95S_{0}$, $0.975S_{0}$ for puts
-
$S_{0}$, $1.025S_{0}$, $1.05S_{0}$, $1.1S_{0}$ for calls.

The hedging exercise is performed over the entire test set, with no overlapping periods. That is, each long straddle position is hedged until expiry, after which a new straddle position is entered.

We use LASSO for the selection of hedging instruments H. To calibrate the $L_{1}$ regularization parameter by examining the in-sample $R^{2}$ and the Mean Squared Error as a function of the penalization parameter for the day on which a new position is entered. We repeat the same procedure every time we enter a new straddle. The values of *α* under consideration are from 0 to 1 in 0.1 increments. LASSO regression is used for instrument selection at time *t* = 0 only. After the hedging instruments have been selected, the hedge ratios are computed via ordinary least squares.

**Figure 23.**
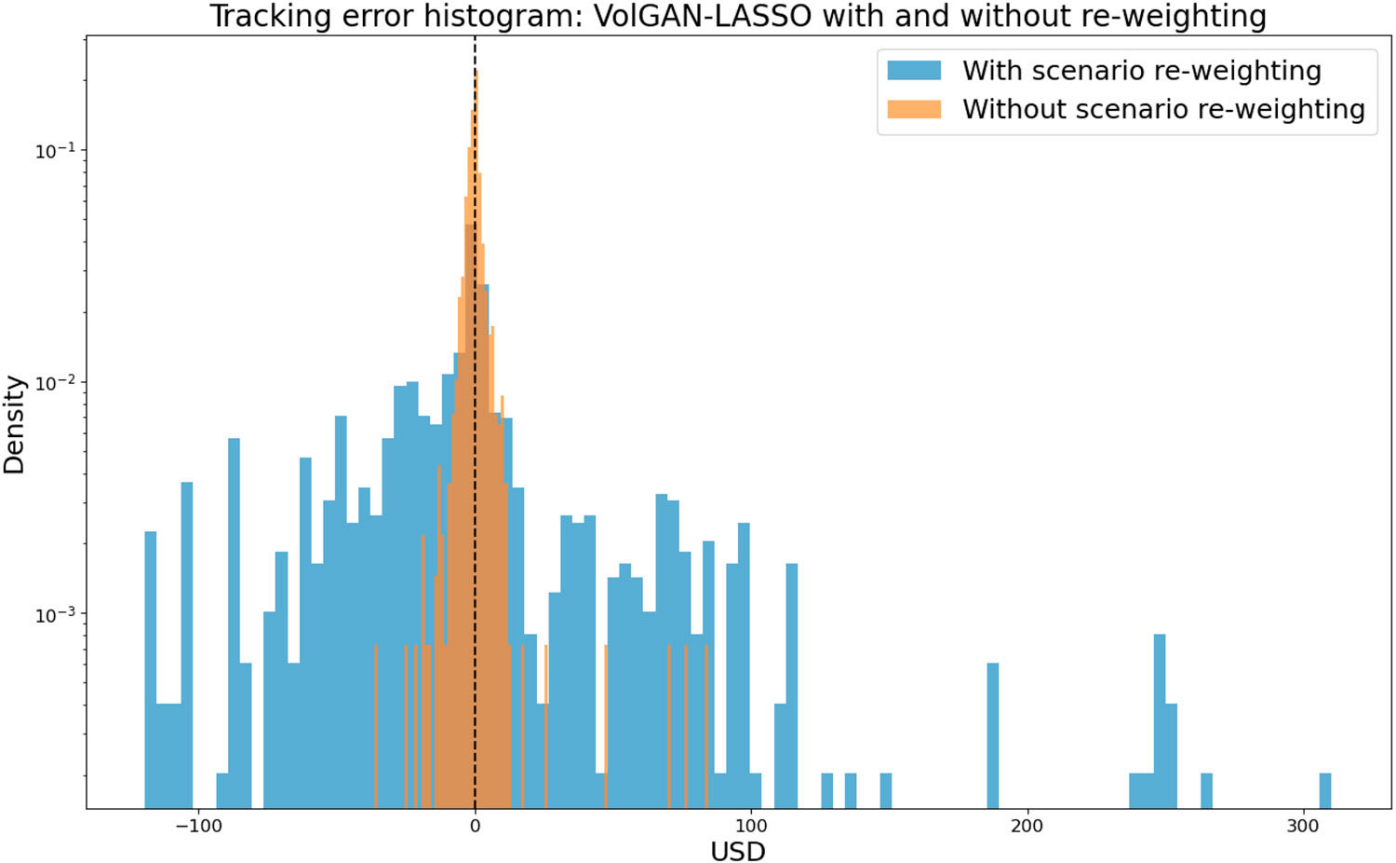
Distribution of tracking error: data-driven hedging via VolGAN and LASSO regression, with and without scenario re-weighting.

We first explore whether scenario re-weighting improves the hedging performance or not. That is, we compare the two LASSO-based methods, using the same values of *α* (chosen using raw outputs). Both methods perform regression using 1000 samples from VolGAN. The tracking error histogram in Figure 23 shows that it is better to use raw VolGAN outputs, since they mimic the market, rather than applying arbitrage-scenario re-weighting. In the remainder of this section, we will focus on raw VolGAN outputs.

Figure 25 shows that the algorithm typically picks 2 or 3 options as hedging instruments during periods of market turbulence, which shows that the portfolio dynamics is well represented by a 2- or 3-factor (implied) volatility process. This result is consistent with the principal component analysis results for VolGAN outputs (Table 4), which show 3 significant factors driving the implied volatility co-movements (Cont and da Fonseca 2002; Cont and Vuletic 2023).

**Figure 24.**
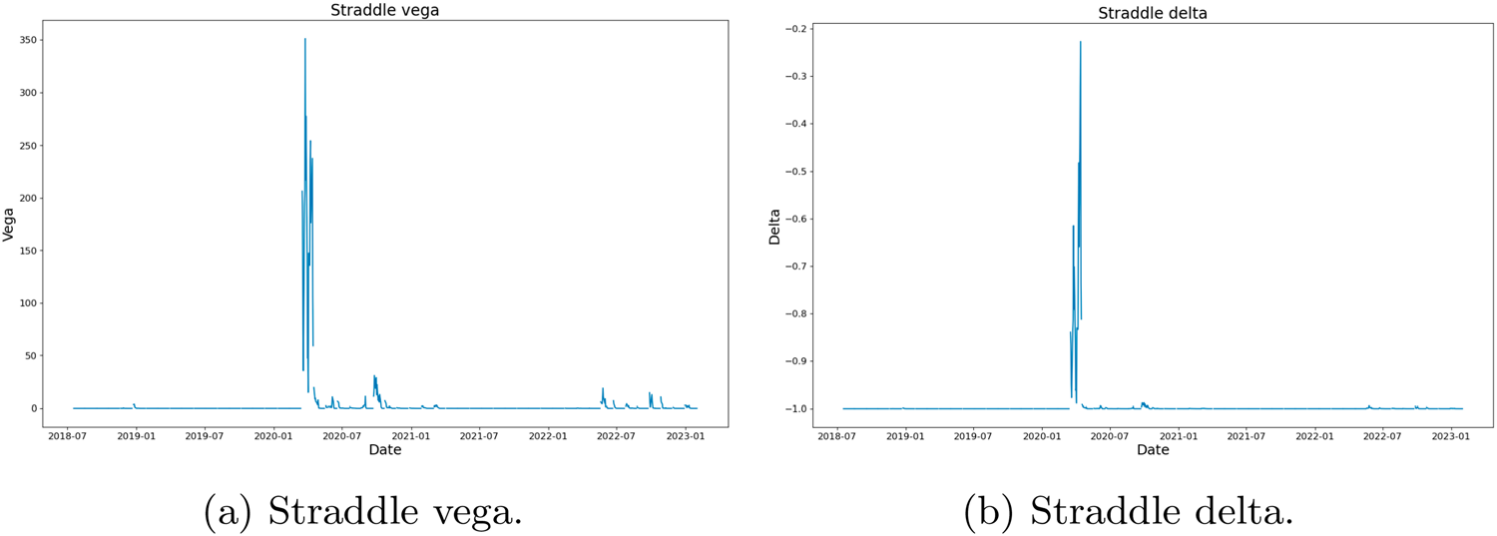
Black–Scholes vega ( $\kappa_{t}^{V}$) and delta ( $\Delta_{t}^{V}$) of the straddle portfolio on the test set. We note a jump in both values at the start of the Covid-19 pandemic. (a) Straddle vega and (b) Straddle delta.

There are periods during which no options are selected for hedging, in line with the straddle vega being zero (Figure 24(a)). Up until the start of the Covid-19 pandemic the straddle delta is equal to minus one (Figure 24(b)), and the straddle vega is zero. The instances with no regularization correspond to 7 options used for hedging.

**Figure 25.**
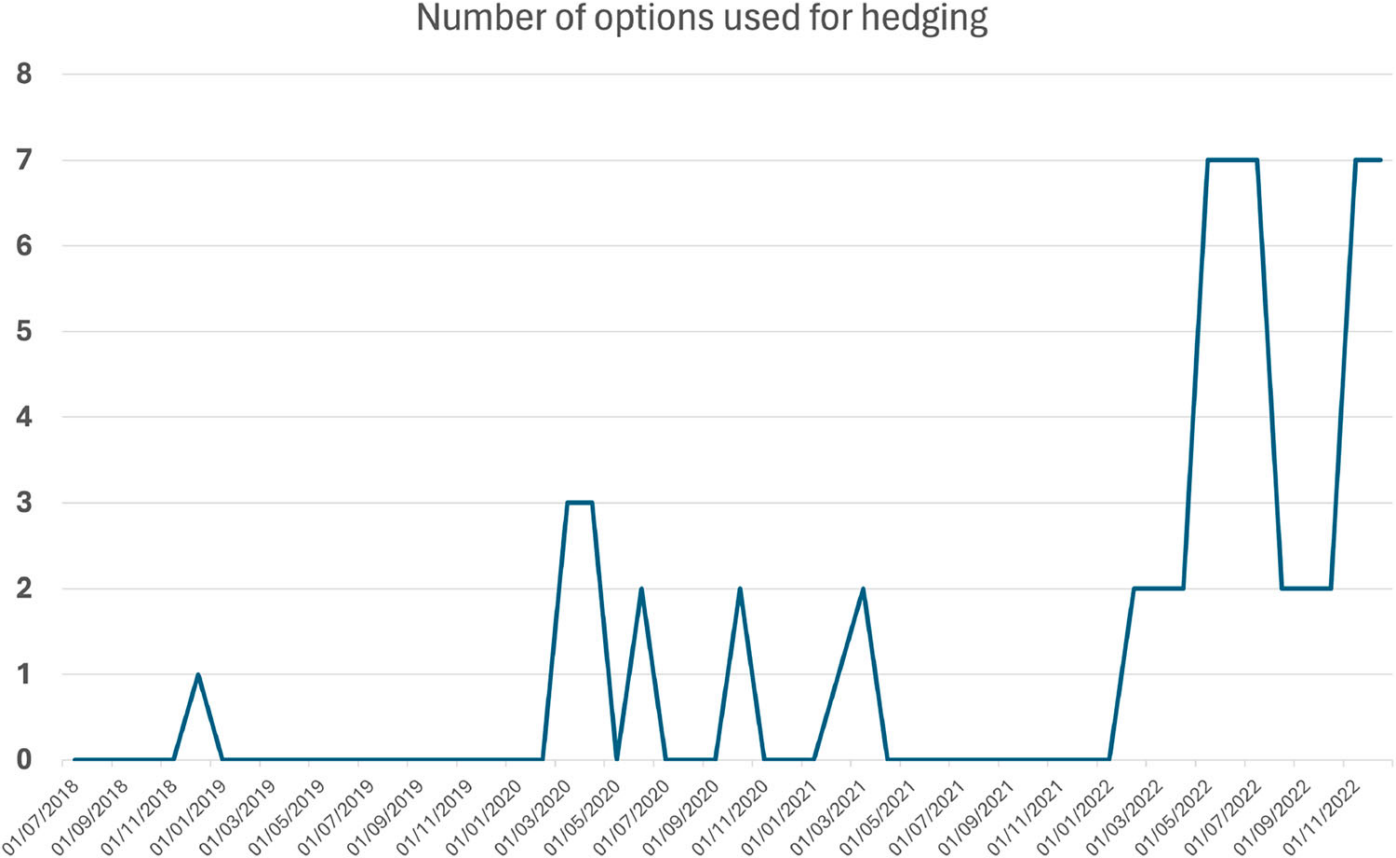
Number of hedging instruments selected using LASSO across VolGAN scenarios. During periods of calm no options are used for hedging. During periods of market turbulence, usually 2–3 options are selected for hedging, indicating that portfolio dynamics is well represented by a 2- or 3-factor (implied) volatility process.

Figure 25 shows that, during the Covid-19 pandemic and the start of Ukraine war, 2–3 options are used for hedging.

Table 10 offers a summary of how many times each option is used for hedging. In all but one instance in which options are used, the call initiated at-the-money was included. The remaining call options were only used in the 5 periods during which no regularization was applied (when α=0 due to our search grid). When a single option was used, it was the at-the-money call.

**Table 10.** Frequency of options selected by LASSO in Methods 3 (no re-weighting) and 5 (with re-weighting).

Option Type	Initial moneyness	Frequency
Put	0.9	6
Put	0.95	7
Put	0.975	16
Call	1	16
Call	1.025	5
Call	1.05	5
Call	1.1	5

These examples illustrate that VolGAN is more flexible than a factor model with a fixed number of factors: the number of effective factors, which corresponds to the number of hedging instruments used, changes dynamically with market conditions.

**Figure 26.**
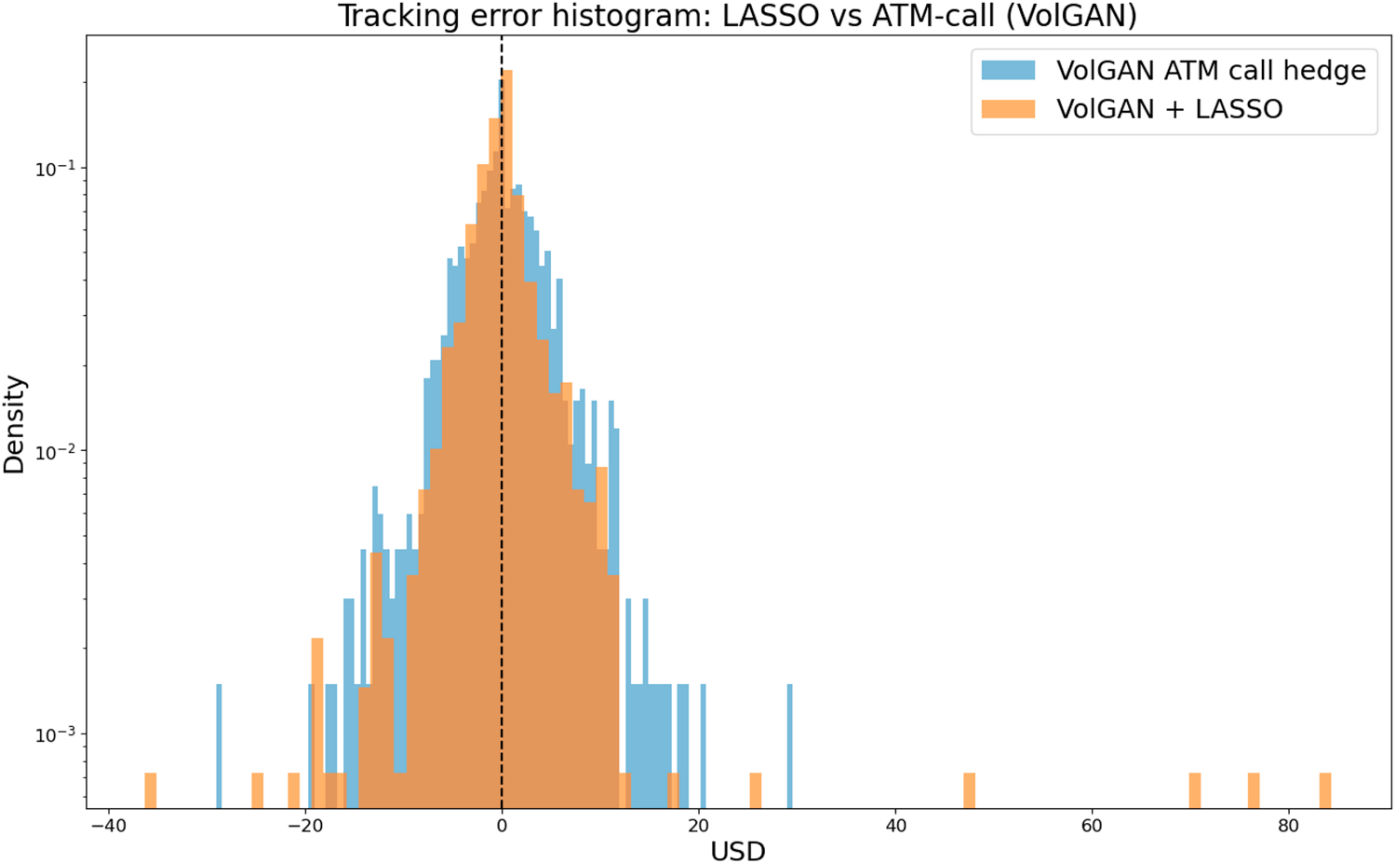
Tracking error distribution: VolGAN + LASSO vs VolGAN at-the-money call hedge.

Before comparing VolGAN-driven methods with delta hedging and delta-vega hedging, we discuss the differences between using the automatic instrument selection via LASSO regression and the call initiated at-the-money. Figure 26 shows the tracking error histograms produced by the two methods. Automatic selection of hedging instruments appears to result in a more concentrated tracking error distribution around zero.

**Figure 27.**
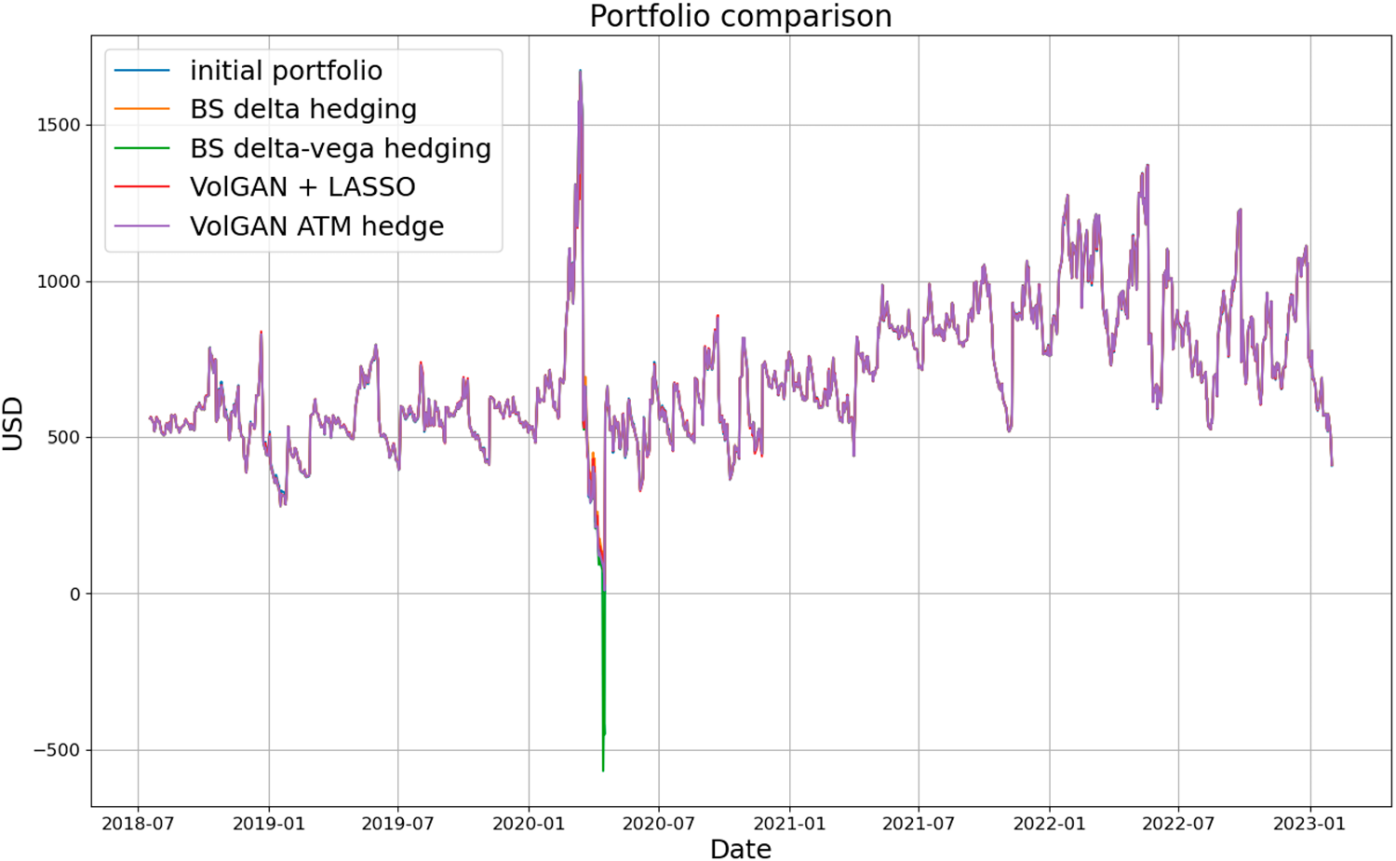
Straddle $V_{t}$ and hedging portfolios ${\hat{V}}_{t}$ corresponding to different methods. Each new straddle position is entered the day after expiry of the previous one.

**Figure 28.**
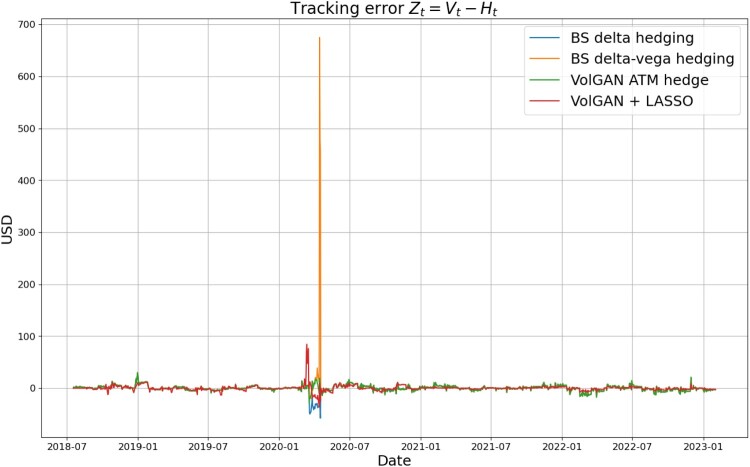
Tracking error $Z_{t}$ corresponding to different methods. Each new straddle position is entered the day after expiry of the previous one.

We compare the two data-driven approaches using raw VolGAN outputs with delta hedging and delta-vega hedging. Figure 27 indicates that away from the initial Covid-19 shock all methods replicate the straddle well. However, during this period, delta-vega hedging shows instability and results in a significant decrease in the hedging portfolio value ${\hat{V}}_{t}$. This does not happen with the other methods, including the VolGAN at-the-money hedge, despite the fact that the two use exactly the same hedging instruments. Such behaviour highlights the fact that the delta-vega hedge ratio corresponding to the option might become unstable, especially if the vega of the option used for hedging becomes small. The corresponding tracking errors $Z_{t}$ as functions of time are displayed in Figure 28.

**Figure 29.**
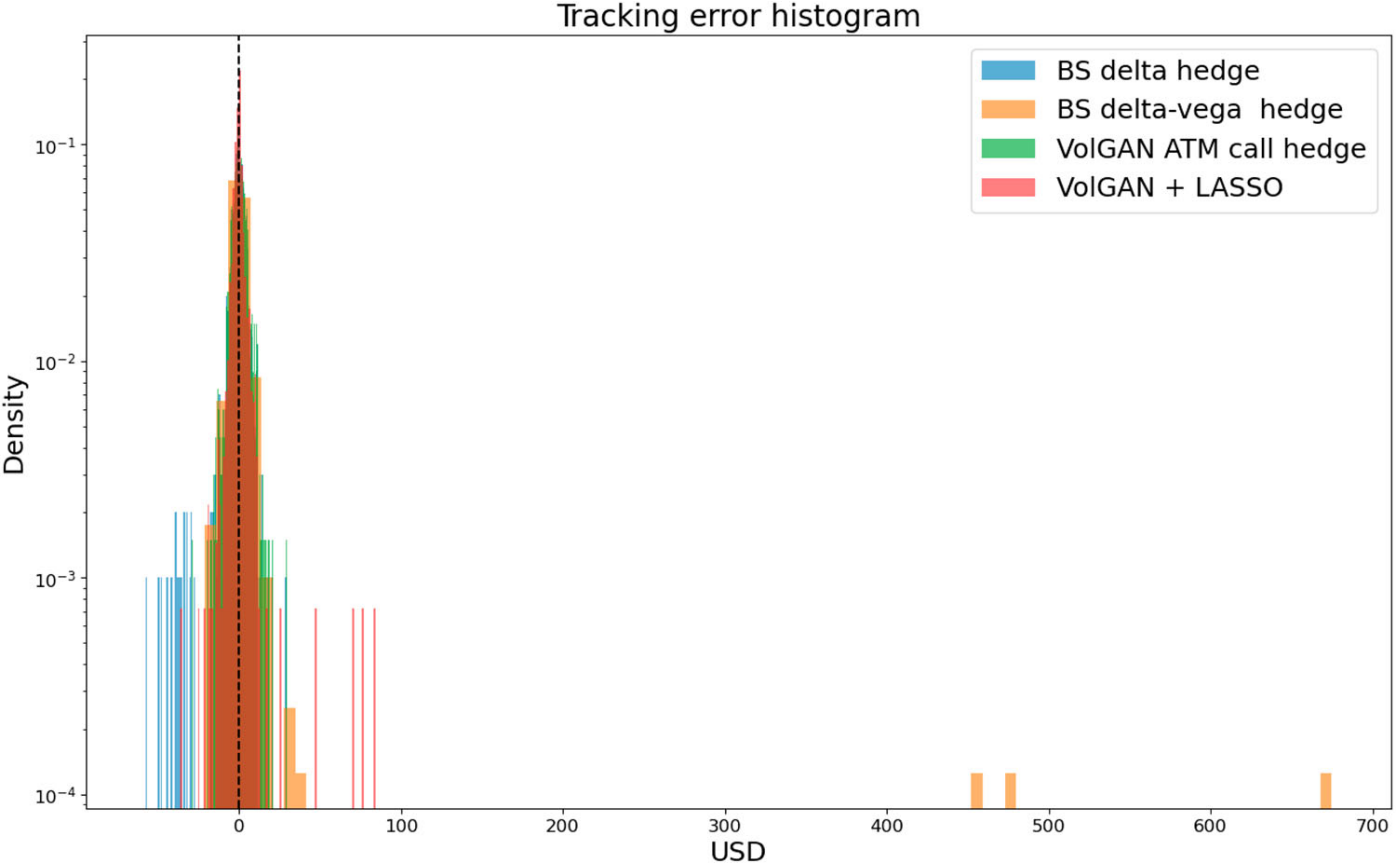
Histogram of the tracking error $Z_{t}$ corresponding to different methods. Each new straddle position is entered the day after expiry of the previous one.

Table 11 contains the tracking error statistics such as mean, standard deviation, and Value-at-Risk ( 5%, 2.5%, 1%) for the Black–Scholes and VolGAN hedging methods. Value-at-Risk (VaR) at level *a* is calculated as the negative *a* quantile of the tracking error. VolGAN + LASSO result in the mean closest to zero, the second lowest standard deviation, and in the lowest Value-at-Risk metrics. The lowest standard deviation is achieved by the ATM hedging via VolGAN. However, comparing the histograms in Figure 26 and the tracking errors in Figure 28, we note that the increase in standard deviation when opting for automatic hedging instrument selection is due to the performance during April 2020. The bulk of the tracking error distribution is slightly tighter for VolGAN + LASSO than for VolGAN + ATM. Delta-vega hedging has lower VaR than delta-hedging, but higher standard deviation, due to the instability evidenced in April 2020. All tracking error distributions are compared in Figure 29. In this instance, data-driven hedging via VolGAN outperforms the Black–Scholes benchmarks. This is particularly impressive given the volatility and the length of the testing period. This test shows that VolGAN is indeed able to capture the co-movements of the implied volatility surface and the underlying.

**Table 11.** Tracking error $Z_{t}$ statistics (mean, standard deviation, 5% Value-at-Risk, 2.5% Value-at-Risk, and 1% Value-at-Risk) as obtained by different models (in USD).

Statistics	VolGAN + LASSO	VolGAN + ATM	BS delta	BS delta-vega
Mean	−**0**.**051**	0.056	−0.614	1.541
St.dev	5.766	**4**.**940**	6.755	28.307
5% VaR	**5**.**815**	7.314	8.310	7.258
2.5% VaR	**8**.**095**	10.692	13.300	10.701
1% VaR	**13**.**172**	13.730	34.023	14.181

## Data Availability

SPX options data is available from OptionMetrics. VIX times series is available from CBOE (www.cboe.com/).
